# The W-Acidic Motif of Histidine Kinase WalK Is Required for Signaling and Transcriptional Regulation in *Streptococcus mutans*

**DOI:** 10.3389/fmicb.2022.820089

**Published:** 2022-04-26

**Authors:** Lingyuan Kong, Mingyang Su, Jiayan Sang, Shanshan Huang, Min Wang, Yongfei Cai, Mingquan Xie, Jun Wu, Shida Wang, Simon J. Foster, Jiaqin Zhang, Aidong Han

**Affiliations:** ^1^State Key Laboratory for Cellular Stress Biology, School of Life Sciences, Xiamen University, Xiamen, China; ^2^Department of Clinical Laboratory, The First Affiliated Hospital of Xiamen University, Xiamen, China; ^3^State Key Laboratory for Oral Diseases and National Clinical Research Center for Oral Diseases, West China Hospital of Stomatology, Sichuan University, Chengdu, China; ^4^Department of Molecular Biology and Biotechnology, The Florey Institute, The University of Sheffield, Sheffield, United Kingdom

**Keywords:** signal transduction, two-component system, histidine kinase, transcriptional regulation, WalR, WalK

## Abstract

In *Streptococcus mutans*, we find that the histidine kinase WalK possesses the longest C-terminal tail (CTT) among all 14 TCSs, and this tail plays a key role in the interaction of WalK with its response regulator WalR. We demonstrate that the intrinsically disordered CTT is characterized by a conserved tryptophan residue surrounded by acidic amino acids. Mutation in the tryptophan not only disrupts the stable interaction, but also impairs the efficient phosphotransferase and phosphatase activities of WalRK. In addition, the tryptophan is important for WalK to compete with DNA containing a WalR binding motif for the WalR interaction. We further show that the tryptophan is important for *in vivo* transcriptional regulation and bacterial biofilm formation by *S. mutans*. Moreover, *Staphylococcus aureus* WalK also has a characteristic CTT, albeit relatively shorter, with a conserved W-acidic motif, that is required for the WalRK interaction *in vitro*. Together, these data reveal that the W-acidic motif of WalK is indispensable for its interaction with WalR, thereby playing a key role in the WalRK-dependent signal transduction, transcriptional regulation and biofilm formation.

## Introduction

Bacteria have adapted to a wide range of dynamic environments, where they have to not only sustain their growth by obtaining sufficient nutrients, but also cope with toxic and ever-changing conditions (Schlegel and Jannasch, 2006). This is particularly apparent for bacterial pathogens, that during infection have to respond to the host to be able to maintain fitness and cause disease (Vouga and Greub, 2016). The inexorable spread of antimicrobial resistance (AMR) makes understanding response mechanisms as potential intervention targets even more pressing (Davies and Davies, 2010).

Two-component systems (TCSs) are major sensing, transduction, and regulatory mechanisms that respond to various intrinsic, environmental, and even toxic stress signals in bacteria (Mascher et al., 2006; Krell et al., 2010). A TCS is generally composed of a sensor histidine kinase (HK) that initiates a histidine autophosphorylation upon stimulation and a cognate response regulator (RR) that is regulated by its respective HK *via* phosphorylation on a conserved aspartic acid residue (Stock et al., 2000; Buschiazzo and Trajtenberg, 2019). A typical RR has a regulatory domain (RD) with the conserved phosphorylatable aspartic acid, and an effector domain (ED) (Gao et al., 2007). Over 60% of the EDs are DNA-binding domains (DBD) that bind specific sets of promoters for transcriptional regulation (Gao et al., 2007, 2019).

Most TCSs are dispensable for bacteria under favorable conditions. However, one TCS, namely, WalRK, also called YycFG or VicRK, is essential for several Gram-positive bacteria, including staphylococci, enterococci, *Listeria*, and streptococci (Martin et al., 1999; Dubrac et al., 2008; Winkler and Hoch, 2008). WalRK is found to be essential among the 13 TCSs in *Streptococcus pneumoniae*, the 14 TCSs of *Streptococcus mutans*, and the 16 TCSs of *Staphylococcus aureus* (Lange et al., 1999; Wagner et al., 2002; Senadheera et al., 2005; Villanueva et al., 2018). WalRK is a master cell wall regulator, and also regulates a variety of vital cellular processes, such as membrane homeostasis, cell division, biofilm formation, and virulence (Ng et al., 2004, 2005; Mohedano et al., 2005; Senadheera et al., 2005; Dubrac et al., 2007; Delaune et al., 2011, 2012; Fukushima et al., 2011; Stipp et al., 2013; Dobihal et al., 2019).

In addition, WalRK is closely involved in the development of antibiotic resistance. Vancomycin is the last resort for methicillin-resistant *S. aureus* (MRSA) strains, but unfortunately, WalRK mutations can arise during vancomycin therapy, which can lead to clinical treatment failure (Jansen et al., 2007; Howden et al., 2011; Shoji et al., 2011; Zhu et al., 2021). TCSs have been hypothesized as antibacterial targets for decades because of their presence in bacteria but absence from mammalian cells (Barrett and Hoch, 1998; Stephenson and Hoch, 2004). The essentiality of WalRK makes it attractive for the design of new antibiotics (Gotoh et al., 2010b; Tiwari et al., 2017). Several groups of compounds targeting WalRK have shown to be effective as antibiotics for further development (Qin et al., 2006; Li et al., 2009; Gotoh et al., 2010a; Eguchi et al., 2011).

*Streptococcal* WalK is shown to be not essential, unlike s*taphylococcal* WalK homolog. They differ in their transmembrane and associated extracellular Cache domains, but are conserved in their intracellular domains (Dubrac et al., 2008; Upadhyay et al., 2016). Like all HKs, WalK has a universal catalytic module that is composed of a conserved dimerization and histidine phosphorylation (DHp) domain and a catalytic ATP-binding (CA) domain [with an HATPase_c fold (El-Gebali et al., 2019)], even though their protein homology is low (Wang et al., 2013). We and others show that such a module of *streptococcal* WalK has constitutive histidine phosphorylation, phosphotransferase, and phosphatase activities (Gutu et al., 2010; Wang et al., 2013). WalR is a typical RR of the OmpR/PhoB family with a DBD of winged helix-turn-helix structure (Okajima et al., 2008; Gao et al., 2019). The RD of WalR is activated by aspartic acid phosphorylation, which regulates its dimerization state (Bent et al., 2004; Gao et al., 2019). Importantly, as a canonical model, the RD of WalR mediates a direct and specific interaction with the DHp domain of WalK mediating aspartic acid phosphorylation and/or dephosphorylation (Skerker et al., 2008; Casino et al., 2009; Podgornaia et al., 2013).

Here, we have investigated the trilateral relationship between WalK, WalR, and promoter DNA. We find that an interesting motif in the WalK C-terminal tail (CTT) is required for WalK to interact with WalR. A conserved residue tryptophan is central in a stretch of acidic amino acids, where mutations disrupted the WalRK interaction, and the competition with its promoter DNA. We have further examined WalK in *S. aureus*, and found that a WD motif is important for the WalRK interaction *in vitro*. Finally, we have shown that mutations of this motif disrupt the phosphotransferase and phosphatase activities of WalK and subsequently biofilm formation of *S. mutans*.

## Materials and Methods

### Protein Preparation

The WalK and WalR coding regions were amplified from *S. mutans* UA159 genomic DNA using PCR. The *walR* gene was cloned into a vector pET-His with *Bam*HI/*Nhe*I sites to create expression constructs pET-His-WalR (1–235), pET-His-WalR (1–136), and pET-His-WalR (129–235) to express N-terminal His-tagged fusion proteins. The same set of expression constructs was made using a vector pET-GST to generate pET-GST-WalR (1–235), pET-GST-WalR (1–136), and pET-GST-WalR (129–235), which were used to produce N-terminal GST-tagged WalR fusion proteins. The *walK* gene fragments (31–450 and 196–450) were cloned into a vector pCRT7 (cloned in *Bam*HI/*Hin*dIII) to express N-terminal His-tagged WalK proteins.

*S. aureus* WalK and WalR coding regions were amplified from *S. aureus* NCTC8325 genomic DNA using PCR. The full-length *walR* (1–233) gene was cloned into pET-GST for an N-terminal GST-tagged fusion protein. A gene fragment encoding WalK (364–608) was cloned into pET-His to express an N-terminal His-tagged fusion protein. All *walK* deletion and site-specific mutations were generated using our modified QuikChange protocol (Mao et al., 2011).

*E. coli* BL21 (DE3) cells transformed with these expression plasmids were grown in Luria-Bertani broth at 37°C to an OD_600_ of ∼1.0 and induced with 0.1 mM isopropyl β-d-1-thiogalactopyranoside (IPTG) for 8 h at 25°C. For His-tagged fusion proteins, the cells from 500 ml of culture were harvested and re-suspended in a lysis buffer (50 mM Tris-HCl, pH 8.0, 500 mM NaCl, 10% v/v glycerol, 20 mM imidazole, and 5 mM β-mercaptoethanol). The supernatant, after a complete lysis by sonication on ice and centrifugation at 40,000 *g* was incubated with Ni^2+^-NTA resin (Millipore, Burlington, MA, United States) for 2 h at 4°C. The agarose beads were collected into a column and washed with the lysis buffer. The proteins were eluted from the beads using an imidazole gradient of 50–500 mM. For GST-tagged fusion proteins, cells from 500 ml of culture were harvested and resuspended in 50 ml of lysis buffer (20 mM Tris-HCl, pH 8.0, 500 mM NaCl, 10% v/v glycerol, 5 mM β-mercaptoethanol, and 1 mM EDTA), and sonicated for 5 min on ice. The lysate supernatants were collected after a centrifugation at 40,000 *g* for 30 min, and mixed with 2 ml of glutathione agarose (50/50 slurry, Thermo Fisher Scientific, Waltham, MA, United States) for 2 h at 4°C. The agarose beads were collected into a column, washed with 10 ml of wash buffer (20 mM Tris-HCl, pH 8.0, 500 mM NaCl, and 10% v/v glycerol) and eluted in the same buffer with additional 20 mM reduced glutathione. The eluate fractions containing the desired proteins were concentrated to ∼1 ml using Amicon filters (Millipore) and further purified by Superdex 200 size exclusion chromatography (GE Healthcare, Chicago, IL, United States). Protein quality and quantity were analyzed in SDS-PAGE and stained by Coomassie brilliant blue (CBB).

### GST Pull-Down Assay

His-tagged and GST-tagged proteins at equal molar amounts (∼3 μl of 50 μM each) were incubated with 20 μl of glutathione agarose beads (50/50 slurry) in 1 ml of binding buffer (20 mM Tris-HCl, pH 8.0, 200 mM NaCl, 5% v/v glycerol, and 2 mM EDTA) for 2 h at 4°C. The beads were collected by centrifugation at 3,000 rpm for 2 min at 4°C after being washed 3 times with 1 ml of ice-cold binding buffer, and mixed with 25 μl of SDS-loading buffer. The supernatants containing all bound proteins were analyzed in 12% w/v SDS-PAGE and stained using CBB.

### Native PAGE and Electrophoretic Mobility Shift Assay

Protein–protein interactions were examined in 8% w/v native PAGE, which uses the same Tris-glycine gel system as a standard SDS-PAGE, but without SDS. Electrophoretic mobility shift assay (EMSA) is another form of native PAGE to examine protein–DNA interactions in the Tris-Borate-EDTA buffer system. A 25-mer DNA oligo (25-mer, GTT TGT TATAAAAG TGT TACAATAC) taken from the *gtfC* gene promoter of *S. mutans* was chemically synthesized and labeled with fluorescein (Sangon, China). Double-stranded DNA was produced by annealing two oligos at 95°C for 5 min and cooled down to RT. DNA oligo at 20 μM was mixed with WalR or DBD and incubated in a binding buffer (20 mM Tris-HCl, pH 7.5, 100 mM NaCl, 50 mM KCl, and 1 mM DTT) for 15 min at RT. WalK 196–450 at various molar ratios to WalR or DBD was directly added into the mixtures and incubated for another 15 min. Before loading onto the gels, 50% v/v glycerol was added and samples were analyzed on an 8% w/v gel, on ice, using 120 V for 1 h. The gel was then visualized by UV for DNA, and further stained by CBB for proteins.

### Isothermal Titration Calorimetry

ITC titration experiments were carried out at 25°C using MicroCal iTC200 system (GE healthcare, Chicago, IL, United States). *S. mutans* WalK WT (196–450), CTT mutants, and the *S. mutans* WalR full-length buffers were exchanged using gel filtration to a buffer of 20 mM Tris-HCl, pH 8.0, 200 mM NaCl, and 20% v/v glycerol. The iTC200 cell was filled with 200 μl of 20 μM WalR protein solution, and its syringe was filled with 41 μl of 500 μM WalK WT and CTT mutants. The delay time was set for 60 s for the first 1-μl injection. The rest of the titrations were completed with 2 μl of ligand per injection for 20 intervals of 120–150 s. Reference power was 5 μcal/s. The stirring speed was 1,000 rpm. All ITC data were processed in Origin 7.0.

### Autokinase Assay

Phosphorylation of WalK was analyzed using an ATPγS-based protocol (Carlson et al., 2010). WalK proteins at 5 μM were incubated with 100 μM ATPγS in a kinase reaction buffer (20 mM Tris–HCl, pH 7.4, 100 mM NaCl, 50 mM KCl, 2 mM MgCl_2_, and 20% v/v glycerol) for 30 min at 37°C, and terminated by 20 mM EDTA. The mixtures were added with 2.5 mM para-nitrobenzylmesylate (PNBM) (ab138910, Abcam, Cambridge, United Kingdom) to alkylate ATPγS for 1 h, at RT. The resulting products were separated by 12% w/v SDS-PAGE and transferred to PVDF membrane at a constant voltage of 100 V for 60 min at 4°C using transfer buffer (25 mM Tris-HCl, pH 8.0,192 mM glycine, and 10% v/v methanol), which was blocked with 5% w/v milk in TBST [20 mM Tris-HCl, pH 8.0, 150 mM NaCl, and 0.05% (v/v) Tween 20] for 1 h, and incubated overnight at 4°C with the first antibody (1:5,000) (Rabbit monoclonal anti-Thiophosphate ester antibody, ab92570, Abcam, Cambridge, United Kingdom). The blot was washed with TBST and incubated for 1 h at RT with the secondary antibody (1:2,500) (Goat anti-rabbit IgG-peroxidase antibody, A154, Proteintech, China). Protein targets were finally developed using chemiluminescence (WesternBright ECL, K12045, Advansta, San Jose, CA, United States) and imaged in Gel Doc XR + System (Bio-Rad, United States).

### Phosphatase Assay by Phos-Tag Gel Mobility Shift

The WalR full-length at 50 μM was phosphorylated for 1 h at 37°C in 50 mM acetyl phosphate (AcP), 50 mM Tris-HCl, pH 7.4, 50 mM KCl, 2 mM MgCl_2_, and 20% v/v glycerol. The phosphorylated WalR was further purified in the ITC buffer above by gel filtration S200. The peak was collected and concentrated down. The mixture was diluted by 10 × in a buffer of 50 mM Tris-HCl, pH 7.4, 50 mM KCl, 2 mM MgCl_2_, and 10 mM ADP, and mixed with WalK at ratio of 1:5 (*S. mutans* WalK/WalR) and 1:2 (*S. aureus* WalK/WalR). The phosphorylation state of WalR at different time points was analyzed by Phos-tag gel mobility shift (PMS) (Barbieri and Stock, 2008). Briefly, regular 8% w/v SDS gels (acrylamide:bis-acrylamide 29:1) were prepared with additional 50 μM Phos-tag acrylamide (Wako, Japan) and 100 μM ZnCl_2_. The gels were run at 120 V for 120 min on ice for the best mobility shift and stained with CBB. Images were processed using the open-source software ImageJ (Fiji package).^1^

### Phosphotransferase Assay

Direct quantification of phosphorylated WalR was difficult due to intrinsic phosphatase activity of WalK. The phosphoryltransferase activity was therefore analyzed by reduction of a phosphorylated WalK. For *S. mutans* WalK/WalR, WalK proteins at 5 μM were autophosphorylated with 100 μM ATPrS in the kinase reaction buffer described above for 30 min at 37°C, and mixed with 25 μM WalR proteins at RT. Reactions were stopped by 20 mM EDTA at designated time points. Phosphorylation states of WalK and WalR were detected by PNBM as described above. For *S. aureus* WalK/WalR, 10 μM WalK proteins were autophosphorylated with 100 μM ATP in the kinase reaction buffer described above for 30 min at 37°C. Phosphorylated WalKs were mixed with 10 μM WalR proteins at RT. Reactions were stopped by SDS-loading buffer at each time point. The resulting products were separated by 10% w/v SDS-PAGE and phosphorylation states were examined by Western blot as described above with the exception that an anti-N1-phosphohistidine (1-pHis) antibody (MABS1352, Sigma-Aldrich) with a dilution of 1:1,000 was used as primary antibody. Images were processed using the open-source software ImageJ (Fiji package).

### *Streptococcus mutans* Mutants

*S. mutans* strains with *walK* mutations were generated by the marker free Cre-loxP method (Biswas et al., 2007), shown in Supplementary Figure 4A. Briefly, an intergenic region between *walK* and *walX* (WalK residue 196-WalX residue 267) was amplified and cloned into the pET-His vector, resulting in pET-His-WalKX196-450-267, which was further engineered with a *Sma*I restriction site for *loxP* insertion and a gene fragment to express a C-terminal Flag-tag in addition to different mutations in WalK. The *Sma*I site was inserted with a *loxP*-kanamycin (loxP-Kan) resistance cassette amplified from pUC4Km. The plasmids were linearized with *Bam*HI and transformed into *S. mutans* UA159 with 50 mM kanamycin selection (Banerjee and Biswas, 2008). A plasmid pCrePA, which transiently expressed Cre recombinase, to excise the chromosomally integrated *loxP*-Kan cassette at 30°C, was then transformed into the clones. The pCrePA plasmid was finally removed by growth at 37°C. The mutations engineered into the *S. mutans* genome were verified by PCR and DNA sequencing. All bacterial strains grew at the same rate as WT (Supplementary Figure 4B).

### Detection of WalR Phosphorylation *in vivo*

*S. mutans* UA159 WT and its mutants were grown at 37°C for 48 h in Todd-Hewitt yeast extract (THYE) liquid medium. Cells from ∼15 ml of culture were harvested by centrifugation at 4°C (6,000 rpm, 6 min), and washed in phosphate-buffered saline (PBS) buffer to completely remove growth medium. Collected cells were either immediately used for the following experiments or frozen in liquid nitrogen and stored at -80°C. Cell pellets were resuspended in a 20-μl PBS buffer with 20% v/v glycerol, 1 mM PMSF, 1 mM pepstatin, and phosphatase inhibitor complex I (Sangon, China), and homogenized in a frozen cold chamber of an Automated Multi-sample Grinder (Jingxin, China) for 90 s at 65 Hz. The supernatants were collected after a centrifugation at 4°C (13,000 rpm, 10 min), and immediately analyzed on ice by 10% w/v SDS-PAGE containing 50 μM Phos-tag acrylamide and 100 μM ZnCl_2_. Proteins were transferred to a PVDF membrane at a constant voltage of 100 V for 90 min at 4°C using a buffer (25 mM Tris-HCl, pH 8, 192 mM glycine, and 10% v/v methanol) in a wet-tank transfer apparatus after electrophoresis. The PVDF membrane was then blocked by the blocking solution (5% w/v BSA, 1 × TBST buffer), and blotted with the anti-WalR antibody generated in rabbits (Supplementary Figure 7), and stained by the secondary antibody (1:2,500) (Goat anti-rabbit IgG-peroxidase antibody, A154, Proteintech, Wuhan, Hubei, China). The phosphorylated vs. non-phosphorylated WalR ratio was estimated from band intensities using the open-source software ImageJ (Fiji package).

### Scanning Electron Microscopy

Overnight cultures of *S. mutans* UA159 WT and mutants were inoculated at 100 × dilution into 2 ml of THYE medium with 1% w/v sucrose in 24-well plates containing 2 × 3 mm square silicon plates, and incubated at 37°C, 5% v/v CO_2_. The culture medium was changed every 24 h. Biofilms were washed with PBS, fixed with 2.5% w/v glutaraldehyde for 30 min, washed again 3 times with PBS, and dehydrated with a series of ethanol (30, 50, 70, 90, 95, and 100% v/v), followed by 100% v/v butanol without any exposure to air. Samples were then lyophilized after being cooled overnight at 4°C and sputter coated with gold for SEM imaging under Gemini field emission scanning electron microscope (Carl Zeiss SUPRA55, Germany).

### Fluorescence Confocal Laser Scanning Microscopy

The sequential development of the biofilm matrix (3D structure) was determined by directly incorporating a fluorescent marker during synthesis of the extracellular polysaccharide (EPS) matrix (Klein et al., 2011). Briefly, dextran labeled with Alexa Fluor 647 (10,000 MW, anionic, fixable, excitation/emission wavelength at 650/668 nm) was added into THYE growth medium. To avoid excessive staining, the working concentration of dextran was adjusted to 1 μM. Before image acquisition, *S. mutans* cells in the biofilm were labeled for 1 h with 2 μM SYTO 9 (green-fluorescent nucleic acid stains, excitation/emission wavelength of 485/498 nm). The plate was incubated in 5% v/v CO_2_ at 37°C with an aluminum foil covered. The imaging was performed using a Zeiss microscope (ZEISS LSM 700, Germany). Four random positions were selected to generate confocal image series by optical sectioning (1,024 × 1,024 pixel for visualization in tagged image file format). All CLSM images and three-dimensional biofilm image stacks were analyzed and built by a computer program COMSTAT (Heydorn et al., 2000).

### Quantitative RT-PCR

Biofilms of *S. mutans* UA159 WT and mutants cultured for 48 h in THYE liquid medium were collected and washed 3 times with PBS to completely remove growth medium. The cell pellets were lysed with 1 ml of RNAiso-Plus (Takara, Japan) and vortexed thoroughly. The lysates were mixed with 200 μl of chloroform by vigorous shaking for 20 s and incubated for 5 min at RT. The clean aqueous phase after a centrifugation at 4°C (12,000 *g*, 15 min) was collected, mixed thoroughly with an equal volume of ice cold isopropyl alcohol, and incubated at −20°C for 10 min. Total RNA was harvested by centrifugation at 4°C (13,500 *g*, 10 min), washed with 1 ml of 75% v/v ethanol, and dissolved in 20 μl of RNase-free water. Quantification of RNA transcripts by GoTaq qRCR mix was performed according to the manufacturer’s instructions (Promega, China). To synthesize cDNA, 5 μl of total RNA at 1 μg/μl was mixed with 4 μl of 5 × reaction buffer, 2 μl of 25 mM MgCl_2_, 1 μl of 10 mM nucleotide mix, 0.5 μl of RNasin ribonuclease Inhibitor, 1 μl of reverse transcriptase, 1 μl of 0.025 μg/μl random primer, and 5.5 μl of nuclease-free water, and incubated at 25°C for 5 min (for annealing), 42°C for 1 h (for extension), and 70°C for 15 min (for inactivation). Quantitative PCR was carried out in 5 μl of cDNA in 25 μl of 2 × qPCR master mix, 1 μl of 10 μM forward primer, 1 μl of 10 μM reverse primer, and 18 μl of nuclease-free water on a Real-Time PCR System SteponePlus (ABI, Thermo Fisher Scientific, Waltham, MA, United States) using the following conditions: 95°C for 2 min, 40 cycles of 95°C for 15 s, and 60°C for 60 s. A dissociation step was added to verify the product specificity. All data were normalized and averaged by quantification of 16S ribosomal RNA. All qPCR primers are listed in Supplementary Table 2.

### Mass Spectrometry

Biofilms of *S. mutans* UA159 WT and mutants cultured at 10-cm plates for 48 h were washed 3 times with PBS, transferred to 1 ml of lysis buffer [50 mM Tris-HCl, pH 8.0, 0.5% v/v Triton X-100, 150 mM NaCl, and protease inhibitor (Amresco)], and homogenized with an Automated Multi-sample Grinder (Jingxin, China). The cell supernatants were collected by centrifugation at 25,000 *g* for 10 min at 4°C and quantified using the bicinchoninic acid (BCA) method. Trichloroacetic acid (TCA) was added to 800 μl of supernatants to achieve a final TCA concentration of 25% w/v. Mixtures were vortexed briefly, and incubated on ice for 3 h. The protein pellets were collected at 25,000 *g* for 10 min, washed 3 times with 500 μl of ice-cold acetone, and dissolved in 150 μl of solubilization buffer (100 mM Tris-HCl, pH 8.5, 8 M urea, and 20 mM DTT) by shaking for 15 min at 37°C. Protein solutions were cooled down to RT, incubated with additional 12 μl of 0.25 M iodoacetamide for 20 min in the dark, and diluted to a urea concentration < 2 M by adding 600 μl of 100 mM Tris-HCl, pH 8.5. Trypsin was added at a 1:100 ratio for an overnight digestion by shaking at 37°C followed by 0.2% v/v formic acid at 4°C. After centrifugation at 25,000 *g* for 20 min, the tryptic peptide supernatants were desalted by STAGEtips and loaded into a TripleTOF 5600 (AB Sciex) mass spectrometer coupled to NanoLC Ultra 2D Plus (Eksigent, Redwood City, CA, United States) HPLC system (Li et al., 2015). Each sample was analyzed in four replicates. The acquired wiff files were searched with Maxquant V1.5 against *S. mutans* serotype C (strain UA159, ATCC 700610) in UniProt. Carbamidomethylation was set as a fixed modification, and N-terminal acetylation and methionine oxidation were set as variable modifications. Proteins were determined by searching a reverse database with false discovery rate at 0.01 for all peptides of minimal 7 amino acids. Trypsin specificity was set as C-terminal arginine and lysine, and a maximum of two missed cleavages was allowed. Quantification in MaxQuant was performed using the label-free quantification (LFQ) algorithm (Cox et al., 2014). Bioinformatical analysis was performed in Perseus (Tyanova et al., 2016). The original LFQ values were transformed by the default formula “log_2_(x).” Three replicates in at least one group were applied to filter for valid values. All missing values were input from a normal distribution by setting a shrink-fit width of 0.3 and down shift of 1.8. A total of 1,236 unique proteins were detected and the expressions of 206 proteins were altered at statistically significant levels (ProteomeXchange ID PXD028893).

### Protein Sequence Alignment and Phylogenetic Analysis

Protein sequences of 14 HKs in *S. mutans* UA159 were downloaded from the MiST 3.0 web server (Gumerov et al., 2020). The core sequences of relatively conserved DHp and CA domains were used in our analysis. The sequences were aligned in Clustal Omega (Sievers et al., 2011) and PROMALS3D (Pei et al., 2008). The CA domains of ∼130 amino acids as the most conserved in HKs (from β2-C-terminus) were used to build a phylogenetic tree using the Neighbor-joining method in Mega7 (Saitou and Nei, 1987; Kumar et al., 2016). The evolutionary distances were computed using the Poisson Correction method (Zuckerkandl and Pauling, 1965). Classification of these 14 HKs was based on six key residues following its catalytic histidine in the DHp domain (Huynh et al., 2010) and its conservation was analyzed by Clustal Omega (Sievers et al., 2011) and presented by WebLogo (Schneider and Stephens, 1990). A structural skeleton of the CA domains was produced in Pro-origami server using a high-resolution structure of *Lactobacillus plantarum* WalK that is 64% identical to its *S. mutans* homolog (PDB 5C93) (Stivala et al., 2011; Cai et al., 2017).

The tails of WalKs in different species were searched using *S. mutans* WalK or *S. aureus* WalK sequences in BLAST (Altschul et al., 1990). Unique sequences were manually collected from GenBank (Benson et al., 2011), aligned in Clustal Omega (Sievers et al., 2011), and configured by the ESPript web server (Gouet et al., 1999).

## Results

### Phylogenetic Analysis of 14 Two-Component Systems in *Streptococcus mutans*

**FIGURE 1:**
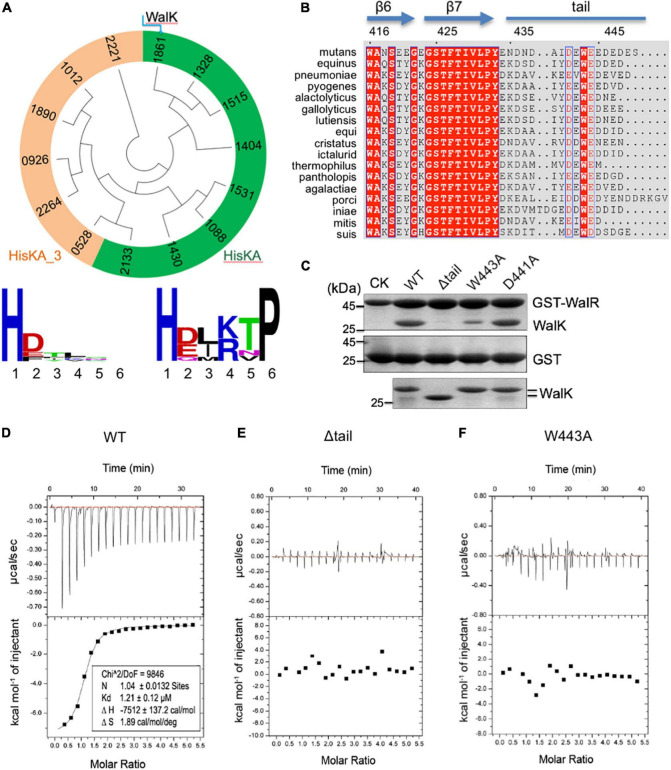
The extended CTT of WalK is unique in *S. mutans* and required for its interaction with WalR. **(A)** Phylogenetic analysis of *S. mutans* HKs. Evolutionary relationship of all 14 HKs is shown in a circular tree, which are grouped into two based on six key residues following their catalytic histidine with conservation for each group below. HKs with a conserved HisKA motif are colored in green. HKs with HisKA_3 motif are grouped in orange. HKs are named with four digits in their protein ID: NP_72****.1. **(B)** Alignment of streptococcus WalK C-terminal sequences. Completely conserved residues are shown in white with a red background and boxed in blue. Highly conserved residues are in red with a white background and boxed in blue. Marked on top are protein secondary structures and residue numbers in *S. mutans*. **(C)** Mutations in the CTT disrupt the WalRK interaction. A GST fusion protein with full-length *S. mutans* WalR was used to pull-down *S. mutans* WalK (196–450) WT and mutant proteins (top gel). As a negative control, GST alone was used to pull down WalK WT and mutant proteins (middle gel). Shown in the bottom gel are 10% of the WalK protein levels used above. CK shows GST-WalR or GST used in the pull-down. **(D–F)** Quantification of the WalRK interaction by ITC experiments. WalK (196–450) WT and mutant proteins were titrated against the full-length WalR, resulting in raw titration curves at the top and their global fittings at the bottom. The derived thermodynamic parameters are shown within.

*S. mutans* genome encodes 12–14 TCSs (Song et al., 2012). There are 14 TCSs in the model strain UA159, with only WalRK having been shown to be essential, as WalK but not WalR can be disrupted (Senadheera et al., 2005; Levesque et al., 2007). To explore the essentiality of WalRK, we searched for unique characteristics of this TCS at the protein level. We collected protein sequences of the 14 HKs of UA159 from MiST database (Gumerov et al., 2020) and aligned their HATPase_c sequences that are the most conserved region in HKs (Supplementary Figure 1A). Even though their identities are only 10.9–38.9%, the structure-assisted approach in PROMALS3D (Pei et al., 2008) produced a sequence alignment of these HKs, which showed the highly conserved motifs within the CA domain, including an N-box, a G1-box, and a G2-box (Supplementary Figure 1B). In contrast, the F-box loop (between the G1-box and the G2-box) appears largely variable in its length and less conserved in sequence. Since this loop plays a key role in ATP binding (Marina et al., 2005; Cai et al., 2017), it is possible that these HKs may bind ATP with different affinity and subsequently differ in autokinase activity. Evolutionary analysis of these HKs using the conserved HATPase_c sequences showed that 8 of 14 HKs, including WalK (HK1861), contain adjacent DHp domains with a HisKA motif HX_1_X_2_X_3_X_4_P, where X_1_ is residues D, E, and Q (Figure 1A). In contrast, the DHp domains of other 6 HKs have a HisKA_3 motif HX_1_X_2_X_3_X_4_X_5_ with residues D, E and F at the X_1_ position. HK1012 with residue F at X_1_ is likely inactive in autokinase activity, as residue F is not able to serve as a general acid in δN protonation (Mechaly et al., 2014; Trajtenberg et al., 2016).

We then analyzed the 14 RRs of the TCSs in *S. mutans*. We collected protein sequences of these 14 RRs from the MiST database (Gumerov et al., 2020), which showed limited identity, from 12.6 to 41.7%. An alignment by PROMALS3D (Pei et al., 2008) revealed two conserved motifs as a β1-DD loop and a β3-D loop in their RDs (Supplementary Figure 2A). The latter D is the phosphorylatable residue D of these RRs, while the former dual DD are key residues in the RD’s active site that coordinate Mg^2+^ or Mn^2+^ for the residue D phosphorylation (Park et al., 2002; Guhaniyogi et al., 2006; Gao et al., 2019). In contrast, significantly less conservation was observed in their EDs. Interestingly, a phylogenetic tree of the full-length RRs clustered 8 RRs, including WalR, that are cognate to the same set of HKs with the HisKA motif, and clustered 6 other RRs that are cognate to the remaining 6 HKs with the HisKA_3 motif (Supplementary Figure 2B).

Together, these protein sequence analyses of 14 *S. mutans* TCSs suggest that WalRK is largely related to 7 other TCSs with the conserved motif HisKA in HKs and to all RRs with the conserved motifs to form an active site. In particular, the HATPase_c domain of WalK is 38.9% identical to CiaH (1515); WalR is 36.3% identical to ScnR (2134), and 41.7% identical to RR1532, both of which are characterized with an effector domain called Trans_reg_C that binds promoter DNA for transcriptional regulations. Therefore, WalRK does not appear unique among the 14 *S. mutans* TCSs at the protein sequence level.

### WalK Has an Extended C-Terminal Tail

From the sequence alignment of HATPase_c domains of 14 *S. mutans* HKs, we observed that WalK (HK1861) has the longest CTT (Supplementary Figure 1B). HK1012 has the second longest tail of 12 amino acids, while WalK has 18 amino acids (433–450). Our previous WalK structure suggested that this CTT is flexible (Wang et al., 2013).

To analyze the CTT of WalK, we aligned WalK C-terminal sequences of 17 streptococcal species (Figure 1B). Even though the tail length varies from 13 to 22 residues, the tail is rich in acidic amino acids D and E that are conserved at positions 441 and 444, wrapping around an invariable W residue at position 443 in the *S. mutans* WalK. Notably, this feature was not found in any other *S. mutans* HKs (Supplementary Figure 1B).

### The Tryptophan in the C-Terminal Tail Is Required for WalRK Interaction

In light of our recent finding concerning the *E. coli* TCS KdpDE, where the CTT of KdpD is required for the signaling of this TCS (Xie et al., 2020), we hypothesized a similar importance for the WalK CTT. Our GST pull-down experiment showed that catalytically active WalK (196–450) was able to readily interact with full-length WalR, but a WalK W443A mutant largely eliminated the interaction, as did a tail deletion mutant Δtail (439–450 deleted) (Figure 1C). In contrast, a D441A mutant did not apparently alter the WalRK interaction. Consistently, the WalRK complex could be observed *via* gel filtration, even in high salt buffers (with 400–800 mM NaCl), suggesting that the interaction is not simply a charge–charge interaction (Supplementary Figure 3).

We further validated the observations by measuring the binding affinity using Isothermal Titration Calorimetry (ITC). Wild-type WalK (196–450) bound the full-length WalR at a *K*_*d*_ = 1.21 μM (Figure 1D). In comparison, the Δtail and W443A mutants led to an undetectable WalRK interaction using ITC (Figures 1E,F).

The biochemical data not only strongly supported the CTT is essential in the WalRK interaction, but also demonstrated that W443 is a key hydrophobic residue, consistent with its high conservation across species.

### The Tryptophan in the C-Terminal Tail Is Required for WalK Signaling

**FIGURE 2:**
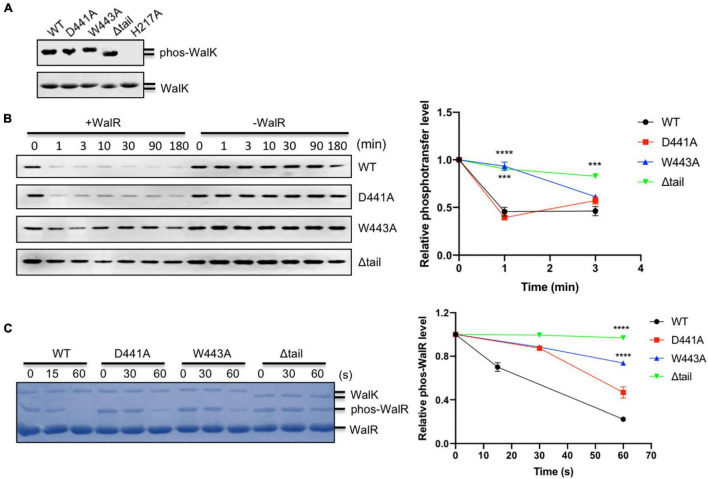
The CTT of *S. mutans* WalK is indispensable for its enzymatic activities. **(A)** Autokinase activity of WalK (31–450) and its tail mutants. The WalK loadings were shown in the lower gel stained by CBB, while the phosphorylation of WalK was detected by ATPγS and anti-thiophosphate antibodies shown in the upper gel. H217A is an autokinase inactive mutant. **(B)** Phosphotransferase of WalK. The phosphotransferase activity was examined by the reduced phosphorylation of WalK incubated with WalR and detected using ATPγS and anti-thiophosphate antibody over time. Quantitative analysis of phosphotransferase activity normalized to 0 min is shown below the gel. **(C)** Phosphatase of WalK. Phosphorylated WalR was incubated with WalK and its derivatives at 1:5 (WalK:WalR), separated from the dephosphorylated WalR in a Phos-tag gel and stained with CBB. Quantitative analysis of phosphatase activity is below the gel. The phosphorylated/dephosphorylated WalR was estimated, normalized to its initial amount at 0 s of the gel. Data presented are means ± standard deviations (error bars) for three independent experiments. Student’s *t*-tests were used to compare mutants to WT at each time point (***p < 0.001 and ****p < 0.0001).

WalK responds to as yet unknown signals to regulate WalR activity through multiple enzymatic activities (autokinase, phosphotransferase, and phosphatase) and so we tested whether the W443 played a role in these activities of WalK. Here, we used the WalK fragment 31–450, containing all intracellular domains, instead of 196–450, in order to better separate it from the full-length WalR in polyacrylamide gels. All WalK CTT mutants D441A, W443A, and a CTT deletion mutant (Δtail) maintained the same autokinase activity as the WT (Figure 2A). We then compared their phosphotransferase activity in the presence of the full-length WalR. The D441A mutant was as active as WT, but the W443A and Δtail mutants significantly lost their activity (Figure 2B).

We examined the phosphatase activity of WalK, by incubation of phosphorylated WalR [phosphorylated with acetyl phosphate (AcP)] with WalK and separation by Phos-tag SDS-PAGE. The phosphatase reaction was able to complete in less than 1 min. While mutation D441 decreased phosphatase activity of WalK, W443 as well as the Δtail mutant significantly disrupted the phosphatase activity of WalK (Figure 2C).

Collectively, these data suggested that W443 in the CTT of WalK is required for the phosphotransferase and phosphatase activities of WalK as it plays an important role in the WalR interaction with WalK. Any mutation in this tail that disrupts the WalRK interaction might impair the signaling processes of this TCS.

### The Tryptophan in the C-Terminal Tail Is Important for the DNA-Binding Domains of WalR and WalK Interaction

**FIGURE 3:**
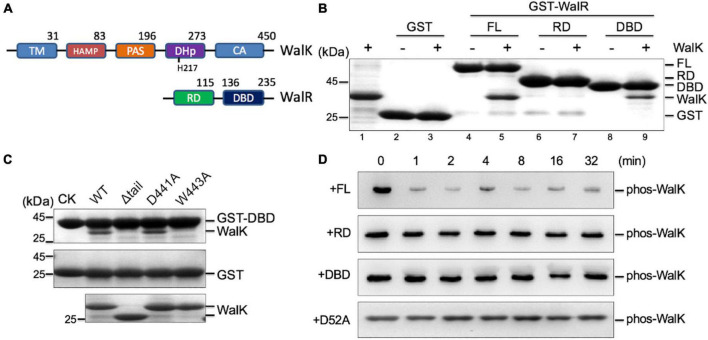
The CTT of *S. mutans* WalK contributes to the interaction with the WalR DBD. **(A)** The domain architectures of WalK and WalR. **(B)** WalK (196–450) interacts with WalR in GST pull-down experiments. The 10% loading controls for WalK, GST, GST-WalR full-length (FL), GST-RD, and GST-DBD are shown in lanes 1, 2, 4, 6, and 8, respectively. **(C)** Mutations in the WalK CTT disrupt the interaction with the WalR DBD. As a negative control, GST alone was used to pull down WalK (middle gel). Shown in the bottom gel are 10% of the WalK protein used above. CK shows GST-DBD or GST used in the pull-down. **(D)** Phosphotransferase activity of WalK is diminished toward the RD of WalR alone. Phosphorylated WalK detected by anti-thiophosphate antibody was incubated with WalR full-length, RD, DBD, and D52A, shown from top to bottom, respectively.

Physical interactions between HKs and RDs of their cognate RRs are believed to be important for the specific phosphorylation flow of TCSs (Laub and Goulian, 2007; Skerker et al., 2008). However, we recently discovered that DBD is also important for signaling and function of the KdpDE TCS in *Escherichia coli* (Xie et al., 2020). To test whether this was also true in the WalRK interaction, we expressed RD and DBD fragments of WalR, and constitutive active WalK (196–450), for a GST pull-down experiment (Figure 3A). We observed that the DBD was able to interact with WalK, but the interaction with the RD was undetectable (Figure 3B). More importantly, we found that W443 is required for the interaction between DBD and WalK because the W443A mutant of WalK was not able to interact with the DBD in a GST pull-down experiment (Figure 3C). Indeed, the RD alone was inefficient in promoting the phosphoryl transfer reaction (Figure 3D), consistent with the *E. coli* KdpDE (Xie et al., 2020).

### The Tryptophan Is Essential for WalK in Competition With Its Promoter DNA

**FIGURE 4:**
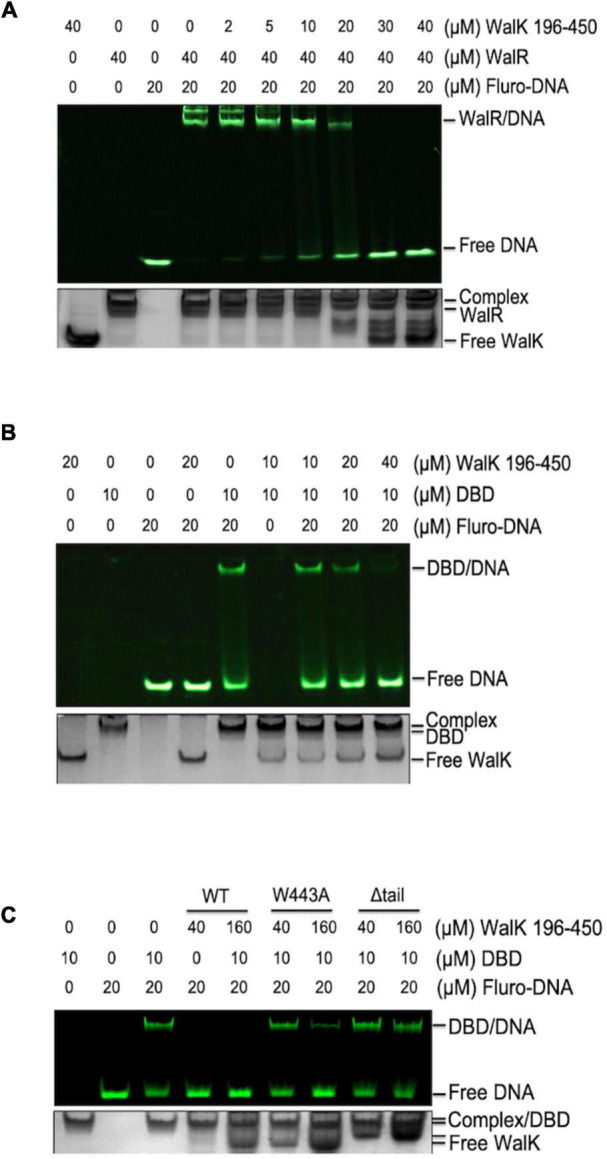
The CTT of *S. mutans* WalK is important for WalK in competition with promoter DNA. **(A,B)** WalK (196–450) competes off fluorescein labeled 25-mer promoter DNA from binding to WalR or DBD in a dose-dependent manner. **(C)** Comparison of the relative ability of CTT mutants (W443A, Δtail) of WalK to compete with DBD in promoter binding. All samples were mixed and incubated for 15 min at RT before loading onto gels. Final concentrations of proteins and DNA used in the reactions were marked above the panels. All EMSA gels were imaged under UV to show DNA in the upper panel and stained with CBB to visualize protein loading in the lower panel.

As DBD directly binds DNA, we asked whether WalK interferes with WalR in binding to promoter DNA regions. WalR binds at specific promoters with two conserved TGT sites (Dubrac et al., 2007). We assessed this issue in an EMSA with fluorescently labeled promoter DNA from the *gtfC* gene, one of the WalKR regulated operons in *S. mutans* (Ayala et al., 2014). Associated with increasing amounts of WalK, the DNA was released from its WalR/DNA complex (Figure 4A). Similarly, WalK was able to compete the DNA off the DBD/DNA complex (Figure 4B). Together, these data suggested that WalK does not directly bind DNA, but competes with the promoter DNA for WalR binding.

The role of the tryptophan in the WalK CTT was then tested. We compared WalK WT with W443A and Δtail mutants in the EMSA experiment. Both mutants had a lower capacity to compete for DNA (Figure 4C). This suggests that a stable interaction of WalRK, promoted by the WalK CTT with W443 as an essential site, contributes to the WalK competition for promoter DNA.

### Low Phosphorylation Levels of WalR Leads to Inefficient Biofilm Formation

**FIGURE 5:**
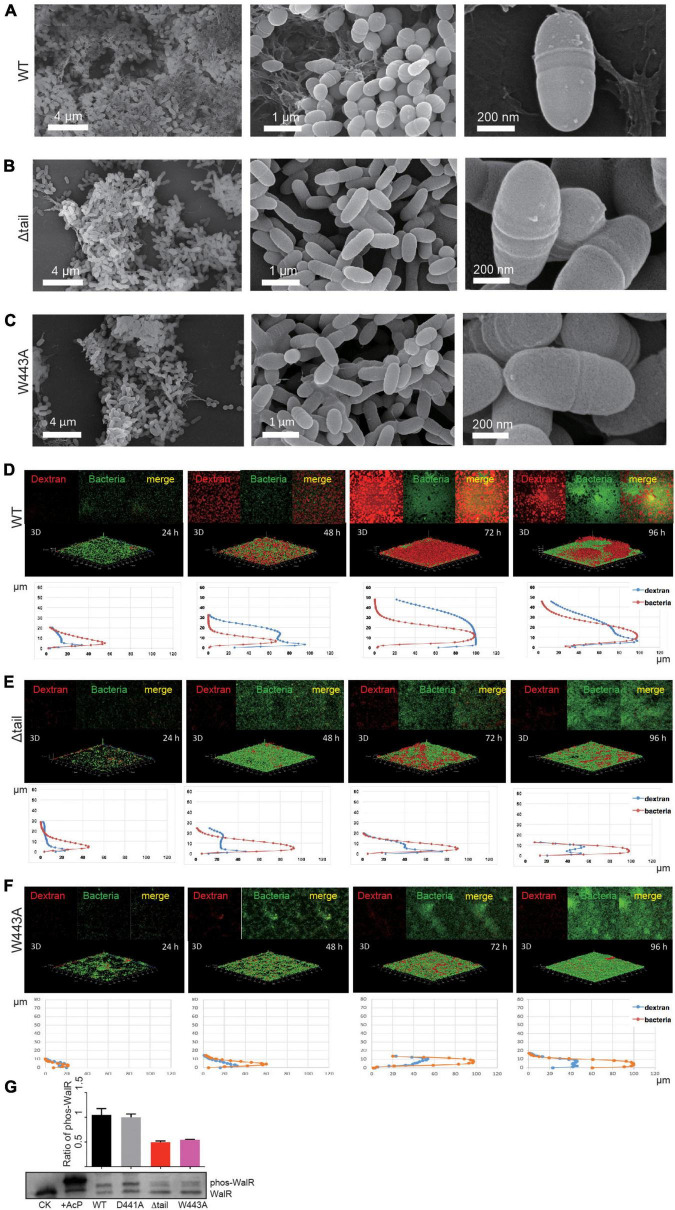
Effect of WalK mutations on biofilm development of *S. mutans*. **(A–C)** SEM analyses of mature biofilms grown for 72 h. **(D–F)** Quantification of biofilms by fluorescent staining. All biofilms were quantified for their thickness and horizontal growth shown below each 3D image. **(G)** Phosphorylation state of WalR *in vivo*. Phosphorylated WalR was separated from its non-phosphorylated state in a Phos-tag gel and detected using an anti-WalR antibody. The non-phosphorylated (CK) and phosphorylated WalR (His-tagged, treated with AcP) were loaded in the first two lanes. The cytoplasmic phosphorylation ratio of WalR shown in the bar chart was determined by band intensities and averaged from three independent experiments with error bars showing standard deviation.

To analyze the role of D441A, W443A, or Δtail alterations to WalK *in vivo*, we generated a set of, otherwise isogenic, *S. mutans* strains bearing the appropriate mutations (Supplementary Figure 4A). All mutant strains had the same growth rate as the WT (Supplementary Figure 4B). We first examined biofilm development of these strains using scanning electron microscopy (SEM). We observed an amorphous thick layer of WT cells embedded in extracellular polymeric substance (EPS) at 48 h (Figure 5A). In contrast, strains with the Δtail deletion or W443A mutation had more disaggregated cells, with lack of apparent EPS (Figures 5B,C).

We next analyzed biofilm formation and dynamics by fluorescence confocal laser scanning microscopy (CLSM). The growth of biofilms was monitored for the entire developmental stages from 24 to 96 h at 37°C. At 24 h, bacterial microcolonies of the WT strain were formed with very little EPS synthesis (Figure 5D). The biofilm was as thin as 18 μm at 24 h and then showed linear growth with a thickness up to 30 μm at 48 h and 45 μm at 72 h (Supplementary Figure 6A). EPS synthesis increased significantly during this period, but reduced at 96 h, indicating that the biofilm possibly completed its growth cycle and had begun to slough. Biofilm dynamics of the D441A strain were similar to the WT (Supplementary Figures 5, 6B). Although bacterial growth appeared to be similar to WT (Supplementary Figure 4B), only isolated colonies of the W443A and Δtail strains formed at 24 h, and less EPS appeared at 48 h (Figures 5B,C). Compared to the WT and D441A, the W443A and Δtail strains showed significantly weaker biofilm formation (Figures 5E,F). The biofilm thickness was only ∼30% of that of the WT strain (Supplementary Figure 6). These biofilms reduced at 96 h without reaching the level of the WT.

To determine a relationship between WalK and WalR phosphorylation *in vivo*, this was examined in the *S. mutans* strains using Phos-tag SDS-PAGE. Unlike that in the WT and D441A strains, phospho-WalR was nearly halved in the W443A and Δtail strains at 48 h (Figure 5G).

### Abnormal Transcriptional Regulation Caused by the Mutations in the WalK C-Terminal Tail

**FIGURE 6:**
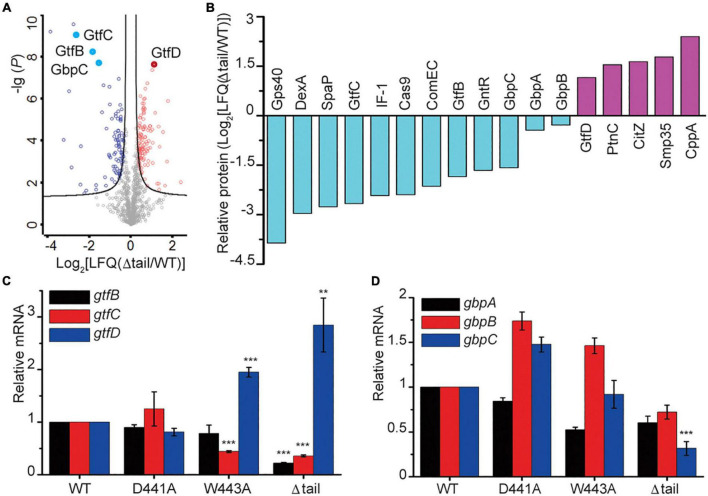
Protein profiling in *S. mutans* biofilms. **(A)** Protein profiling of *S mutans* WT and Δtail strains from a quantitative mass spectroscopy experiment. The *x*-axis indicates the fold change of LFQ in the Δtail strain. The *y*-axis of log (*P*) indicates a significance level of the *t-*test. The black curves separate those proteins at a level of false discovery rate (FDR) = 0.01 and minimal fold changes (S0) = 0.1. Below the curve in gray are those unchanged proteins. Those proteins that were upregulated are colored in red, and those that were downregulated are colored in blue. Total and altered proteins are listed in MS Dataset. **(B)** Proteins that were most altered in the Δtail strain. Proteins were selected at a difference cutoff of > 1.5 except GbpA and GbpB. Five short, functionally unknown, peptides were excluded. The functional annotations of the proteins are shown in Supplementary Table 1. **(C,D)** qRT-PCR analyses of key genes known to be regulated by WalRK. Transcriptional profiles of the genes *gtfBCD* and *gbpABC* were normalized to 16S RNA. Data presented are means ± standard deviations (error bars) for three independent experiments. Student’s *t*-tests were used to compare Δ*tail* strain to *WT* strain (**p < 0.005 and ***p < 0.001).

To determine the consequences of the alterations to the WalK tail at the protein level, we compared the cellular proteome of the WT and Δtail strains by quantitative mass spectrometry (MS) analysis. We detected 1,236 unique proteins, which equate to 62% of the 1963 predicted ORFs in *S. mutans*. The expression of 206 proteins was altered to a statistically significant level between WT and Δtail (Figure 6A). The largest changes (> 2 × LFQ) were found for 39 proteins involved in peptidoglycan metabolism, secreted antigens, competence, and biofilm formation factors (Supplementary Table 1). As shown in Figure 6B, the cell morphology regulator Gps40 was reduced by ∼14×. Two adhesion proteins dextranase and SpaP were reduced by ∼7×. GtfB and GtfC protein levels were reduced by 3–6×, while GtfD was increased by ∼2×. No significant changes were observed for the glucan binding proteins GbpA and GbpB but there was a 3 × reduction of GbpC in the Δ*tail* strain.

Using the proteins with altered levels as a guide, we next determined the role of the CTT in WalK transcriptional regulation. The transcription of the glucosyltransferases *gtfBCD* was determined by quantitative reverse transcription PCR (qRT-PCR). The *gtfB* and *gtfC* transcript levels were reduced by ∼5 × and 3 × in the Δ*tail* deletion strain when compared to the WT and D441A strains, while *gtfD* had a 2–3 × increase (Figure 6C). In the W443A strain, the expression of *gtfC* and *gtfD* but not *gtfB* changed similarly to the Δ*tail* strain. We next quantified the expression of three glucan-binding protein encoding genes *gbpABC*. Only *gbpC* showed a 3 × reduction in the Δtail strain but not in the W443A (Figure 6D).

### A Minimal WD Motif in the *Staphylococcus aureus* WalK C-Terminal Tail Is Indispensable

**FIGURE 7:**
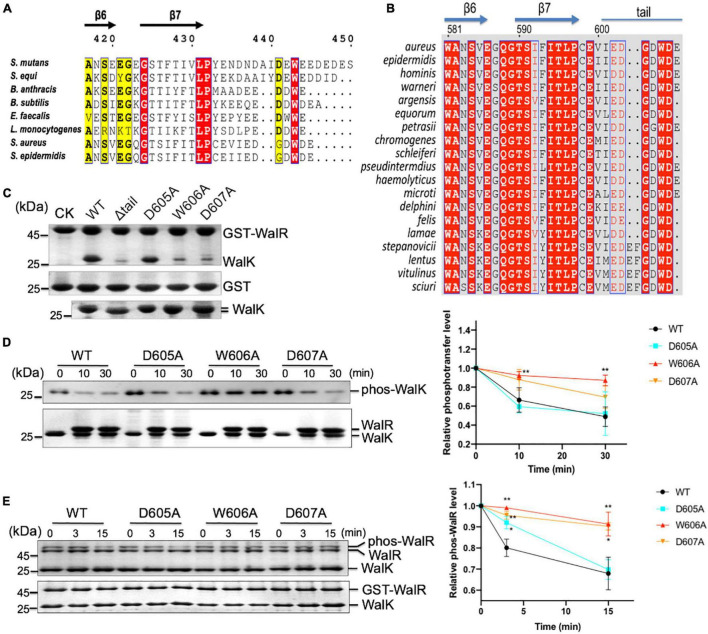
The CTT of WalK is required for *S. aureus* WalRK interaction and enzymatic activity. **(A)** Conservation of the WalK CTT across Gram-positive bacteria. Representative sequences of WalK C-termini were aligned and boxed. The residues are numbered according to *S. mutans* WalK. Completely conserved residues are colored in white in a red background. The less conserved residues are highlighted in yellow. **(B)** Alignment of staphylococcus WalK tail sequences. Completely conserved residues are shown in white in a red background and boxed in blue. Highly conserved residues are in red in a white background and boxed in blue. Marked on top are protein secondary structures and residue numbers in *S. aureus*. **(C)** Mutations in the CTT disrupt WalRK interaction. A GST fusion protein with full-length *S. aureus* WalR was used to pull-down *S. aureus* WalK (364–608) WT and mutant derivatives shown in the top panel. As a negative control, GST alone was used to pull down WalK WT and its mutants shown on the center panel. Shown in the bottom panel are 10% levels of WalK proteins used in each lane. CK shows GST-WalR or GST used in the pull-down. **(D)** Phosphotransferase of WalK (364–608) was disrupted by mutations in the WalK CTT. The phosphotransferase activity was represented by the reduction in WalK phosphorylation relative to T0, the initial phos-WalK. After the addition of WalR, the mixtures were incubated for the indicated time and stopped by the addition of SDS-loading buffer. Loading controls are shown in the bottom gel. The enzymatic activity was quantified shown below. **(E)** Phosphatase of WalK (364–608) was disrupted by mutations in the WalK CTT. Phos-tag gel is shown in the top, and a regular SDS-PAGE below shows the total protein used. The phosphatase activity was quantified by the reduction in the phos-WalR relative to the amount of T0, the initial phos-WalR. The reactions were incubated for the indicated time and stopped by the addition of SDS-loading buffer. Data presented are means ± standard deviations (error bars) for three independent experiments. Student’s *t*-tests were used to compare mutants to WT at each time point (**p < 0.005 and *p < 0.05).

As WalRK is a conserved TCS in several Gram-positive bacteria, we wanted to examine the importance of the WalK CTT in other low GC Gram-positive bacteria. An initial sequence alignment of 8 species from 5 genera showed a variation of the tail length, suggesting that the length is not an important feature of the WalK CTTs (Figure 7A). For example, WalK in *S. aureus* is 5 amino acids shorter than *S. mutans*. However, all these CTTs are rich in acidic amino acids surrounding an invariant tryptophan residue.

To further analyze WalK in *S. aureus*, we aligned WalK C-terminal sequences from 19 staphylococcal species (Figure 7B). Residues G604 and D607 are completely conserved in addition to W606 (numbered according to *S. aureus*). D605 is also conserved in *S. aureus* and the majority of other species. We next mutated these residues to examine their role in a GST pull-down experiment using recombinant protein (Figure 7C). An *S. aureus* WalK (364–608, containing DHp and CA domains) was able to interact with full-length *S. aureus* WalR (1–233), but a tail deletion mutant Δtail (the last 7 amino acids deleted) was not. Single site-directed mutations W606A and D607A, but not D605A, clearly disrupted WalK binding to WalR. These data demonstrate that the WD motif in the WalK CTT is important for the WalRK interaction in *S. aureus*.

The role of the WD motif in WalK activity was then tested. Compared with *S. mutans* WalK, the *S. aureus* WalK showed much slower kinetics of phosphotransferase and phosphatase activities. WalK W606A and D607A mutants, but not D605A mutant, had reduced phosphotransferase activity by nearly 2 × at 30 min (Figure 7D). Also, W606A and D607A had impaired the phosphatase activity of WalK significantly (Figure 7E). Thus, the W residue and its adjacent D residue are required for the WalRK interaction and their phosphorylation processes, consistent with that in *S. mutans* WalK.

## Discussion

As one of the main signaling systems in bacteria, TCSs are responsible for the regulation of a variety of cellular processes and stress responses. The two components of most TCSs interact at a *K*_*d*_ in the low micromolar range, but this is sufficient to determine their cognate relationship *in vitro* (Yoshida et al., 2002; Willett et al., 2013). Indeed, the WalR of *S. mutans* interacts to its WalK with an affinity of *K*_*d*_ = 1.2 μM (Figure 1D). Global coevolution analyses have identified sets of residues in DHp and RD that are important for their specific recognition profiles (Skerker et al., 2008; Weigt et al., 2009), which are supported by several structures of complexes (Varughese et al., 2006; Casino et al., 2009; Trajtenberg et al., 2016). However, the DBD of WalR is involved in the interaction with WalK (Figure 3B), suggesting that an additional layer of interaction may enhance their specific relationship. Phosphotransfer to the RD is clearly inefficient when the DBD is not present (Figure 3D). Similarly, a DBD has recently been shown essential for other TCSs (Li et al., 2010; Xie et al., 2020). Moreover, a crystal structure of KdpDE reveals a non-canonical interface between the DBD and CA domain in addition to the RD and DHp domain, providing a solid explanation for the KdpD competition with promoter DNA (Xie et al., 2020). Therefore, a full-range interaction mediated by both the RD and DBD of WalR is required for the function of WalRK, a unique characteristic of this essential TCS in Gram-positive bacteria. Whether such an interaction model is also applied to other TCSs remains to be investigated.

### Dual Interactions by DNA-Binding Domains Lead to a Competition Between WalK and Promoters

Over 30,000 RRs have been identified from nearly 1,000 bacterial genomes, 66% of which may bind promoter DNA and serve as transcription factors (Ulrich and Zhulin, 2010). Half of these RR transcription factors belong to the OmpR/PhoB family with a structure of a winged helix-turn-helix (wHTH)—three α helices followed by two β strands (Gao et al., 2007). The second helix of wHTH is enriched with several basic residues that are important in binding to the DNA major groove, while a loop between the β strands binds to the DNA minor groove (Blanco et al., 2002; Rhee et al., 2008). We show that the DBD of WalR not only binds DNA but also interacts with WalK. Since WalK does not bind DNA, this result leads to the phenomenon whereby WalK binds the DBD and competes WalR off its promoter DNA (Figure 4).

The competition between an HK and DNA has been observed in the TCS EnvZ/OmpR, where promoter DNA binds phosphorylated OmpR in a competition with EnvZ and inhibits the phosphatase activity of EnvZ (Qin et al., 2001). Phosphorylated OmpR has been shown to bind promoter DNA with a 10 times higher affinity than non-phosphorylated OmpR (Yoshida et al., 2006; Sadotra et al., 2021). In our experiments, non-phosphorylated WalR or DBD also bind to DNA. However, this comparatively weaker interaction can be competed off by WalK. This has also been seen in the KdpDE TCS, where KdpE utilizes an overlapping interface for both KdpD and DNA, leading to a competitive interaction (Xie et al., 2020). The competition was diminished when phosphorylated KdpE was used (Xie et al., 2020). It is possible that when the conditions are optimal for cell growth, high-level phosphorylated WalR enhances a DNA/WalR complex formation and signals get actively transduced. Conversely, when cells are in unfavorable conditions, where the cellular ATP level is low, and ADP level is high, WalK efficiently dephosphorylates WalR that is released from its promoter DNA by a direct binding. Such efficient enzymatic activities of WalK augmented by the DBD possibly keep the free phosphorylated WalR appropriate to bacterial growth conditions. However, whether the competition occurs *in vivo* requires further investigation as WalK localization is also highly regulated in cells (Gutu et al., 2010; Fukushima et al., 2011; Poupel et al., 2016).

### WalRK Regulation in Biofilm Formation

Biofilm formation is a common phenotype of pathogens like *Streptococci* and *Staphylococci*. In *S. mutans*, WalRK has been shown to regulate a set of genes involved in biofilm formation, including *gtfBCD*, *gbpB*, and *ftf* (Senadheera et al., 2005). Our data revealed that production of GtfBCD and GbpC was significantly altered in Δ*tail* mutant with upregulation of GtfBC and GbpC and downregulation of GtfD levels (Figure 6). Interestingly, lower phosphotransferase and phosphatase activities of WalK mutations did not lead to dramatic downregulation of all WalR-regulated genes (Supplementary Table 1). This is consistent with the fact that WalK is not essential in *S. mutans*. Another possibility is that other co-regulators like GcrR and VicX may compensate the loss of WalK activity. It has been reported that GcrR can be phosphorylated by WalK and cross-regulate *gtfBC* with WalR (Downey et al., 2014). VicX is the last gene in the *S. mutans wal* operon that was proposed to co-modulate *gtfCD* and *gbpB* expression with the WalRK system (Lei et al., 2015).

### The Contribution of the WalK C-Terminal Tail to Its Essentiality of WalRK

As the only essential TCS that is currently known in several Gram-positive bacteria, WalRK regulates a diverse of cellular processes in addition to stress responses (Dubrac et al., 2008). Even though its role is largely centered on homeostasis of the bacterial cell wall, what makes it essential remains elusive. WalR is a typical OmpR family member (Supplementary Figure 2), and thus we focused our attention on WalK, its cognate sensor. WalK is the only PAS domain-containing HK in the *S. mutans* UA159 strain. An extracellular CACHE domain is found in all WalK homologs of other bacteria except streptococci. The CACHE and PAS domains may contribute to essentiality by sensing signals that are yet to be fully identified, for example, peptidoglycan fragments or cellular zinc concentration (Dobihal et al., 2019; Monk et al., 2019). The fact that *S. mutans* WalK does not have the periplasmic CACHE sensory domain, while the intracellular PAS domain exists in several other *S. aureus* HKs, suggests that both CACHE and PAS domains are likely not essential elements for WalK.

Interestingly, WalK has the longest CTT in *S. mutans* (Supplementary Figure 1). Even though it is shorter in *S. aureus*, WalK homologs have a characteristic WD motif (Figure 7B). Our *in vitro* and *in vivo* data have shown that such a highly conserved tryptophan is required for WalRK signaling and function, similar to a W-acidic motif that is conserved in adaptors of the kinesin light chain (Pernigo et al., 2013). Recently, we have shown that a CTT extension of KdpD is important for the KdpDE interaction and signaling, but the CTT does not have a conserved tryptophan residue that is essential for its function (Xie et al., 2020). Unlike WalRK, however, KdpDE is only activated under conditions of low intracellular K^+^ and is non-essential for Gram-negative bacteria.

In addition to unique sensor characteristics, WalK is a focus that orchestrates the expression of a network of components that are important for bacterial metabolism and virulence (Poupel et al., 2018; Wu et al., 2019), as well as essential components of cell division (Fukushima et al., 2011; Stamsas et al., 2017; Zucchini et al., 2018). Such a focal activity renders WalRK as a target for antimicrobial development. Therefore, mechanistic understanding paves the way for rational design of interventions to control a range of Gram-positive AMR pathogens.

## Data Availability Statement

The datasets presented in this study can be found in online repositories. The names of the repository/repositories and accession number(s) can be found below: http://www.proteomexchange.org/, PXD028893 (Ma et al., 2019).

## Author Contributions

LK, MS, and JS completed most experiments and analyzed the data. MX, YC, and JW initiated *in vitro* experiments. MW, SH, SW, and JZ established *in vivo* assays. LK, SF, and AH designed experiments, analyzed data, and finalized the manuscript. All authors contributed to the article and approved the submitted version.

## Conflict of Interest

The authors declare that the research was conducted in the absence of any commercial or financial relationships that could be construed as a potential conflict of interest.

## Publisher’s Note

All claims expressed in this article are solely those of the authors and do not necessarily represent those of their affiliated organizations, or those of the publisher, the editors and the reviewers. Any product that may be evaluated in this article, or claim that may be made by its manufacturer, is not guaranteed or endorsed by the publisher.

## Footnotes

1
https://rsb.info.nih.gov/ij/
