# Supplementary material for: The W-Acidic Motif of Histidine Kinase WalK Is Required for Signaling and Transcriptional Regulation in Streptococcus mutans
Source: Front Microbiol. 2022 Apr 26;13:820089. doi: 10.3389/fmicb.2022.820089 (PMC9087282; doi:10.3389/fmicb.2022.820089)
Supplement: Supplementary file 1 [file Data_Sheet_1.docx]

Supplementary figure 1

A


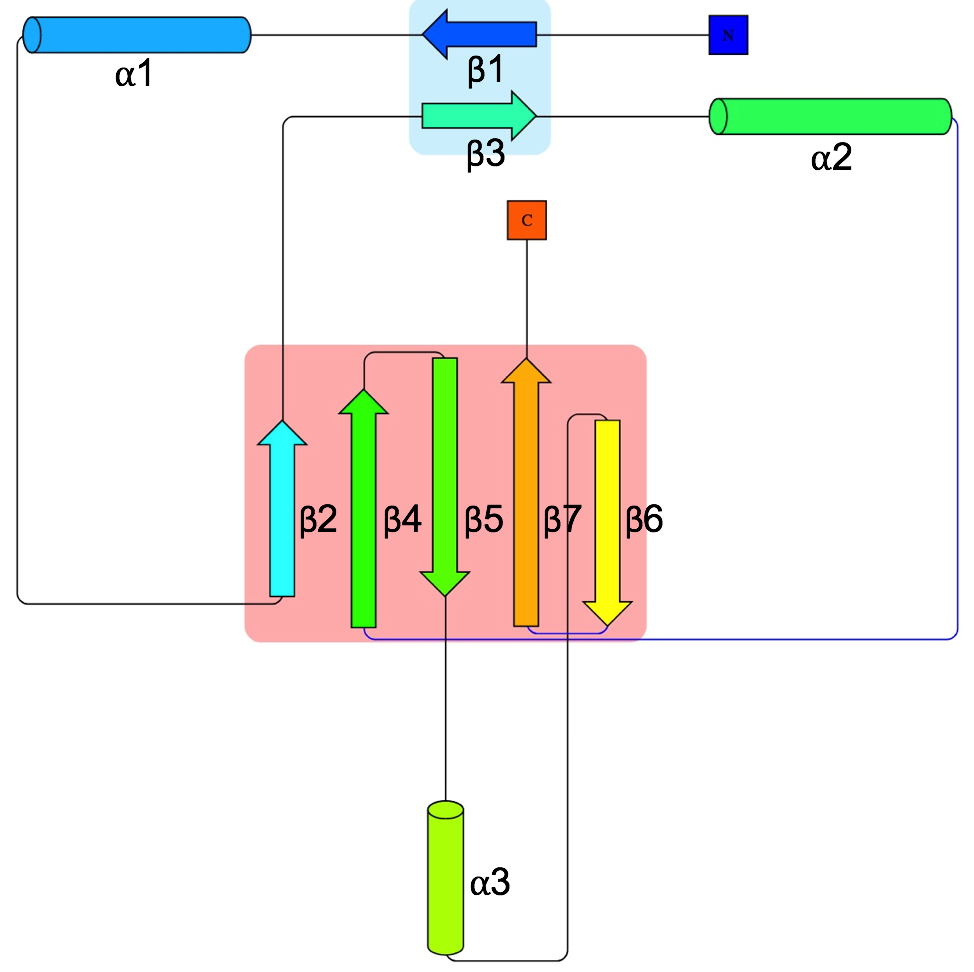


B


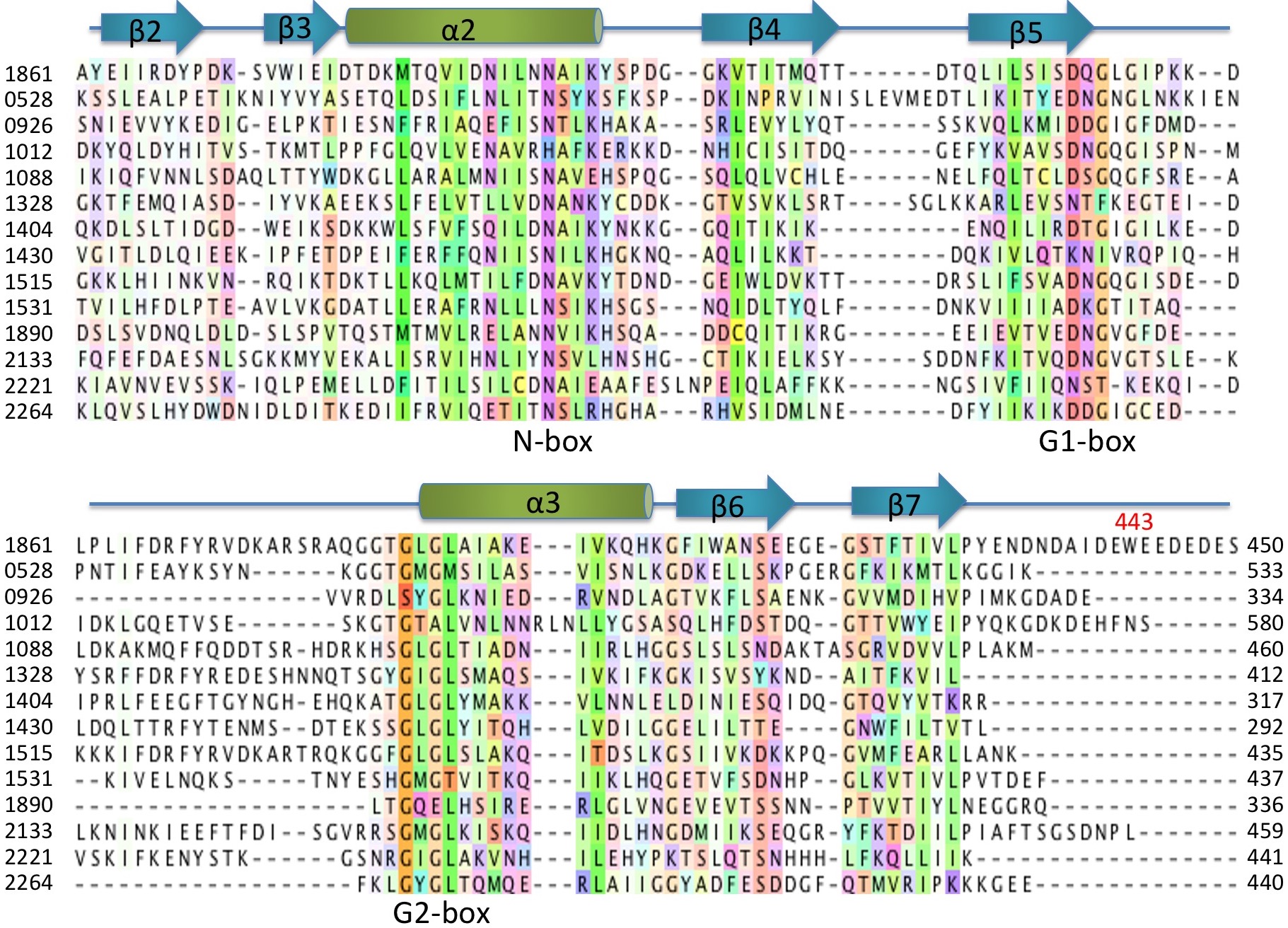


Figure S1. Sequence alignment of the CA domains of 14 HKs in *S. mutans*.

(A) Conserved structural skeleton of CA domain of HKs. The skeleton was produced by the Pro-origami server using a high-resolution structure of *Lactobacillus plantarum* WalK that is 64% identical to its *S. mutans* homolog (PDB 5C93) (Cai et al., 2017; Stivala et al., 2011). The HATPase_c fold from β2 to C-terminus, including α2 and α3, is highlighted in pink. (B) HATPase_c protein sequences of ~130 amino acids were aligned in PROMALS3D (Pei et al., 2008). Residues are colored progressively by their conservations in Jalview (Waterhouse et al., 2009). N-, G1-, and G2-box are the conserved motifs of these CA domains. HKs are named with four digits in their protein ID: NP_72****.1. Total protein length of each HK is marked at end of the alignment. Trp443 is highlighted in red.

Supplementary figure 2

A


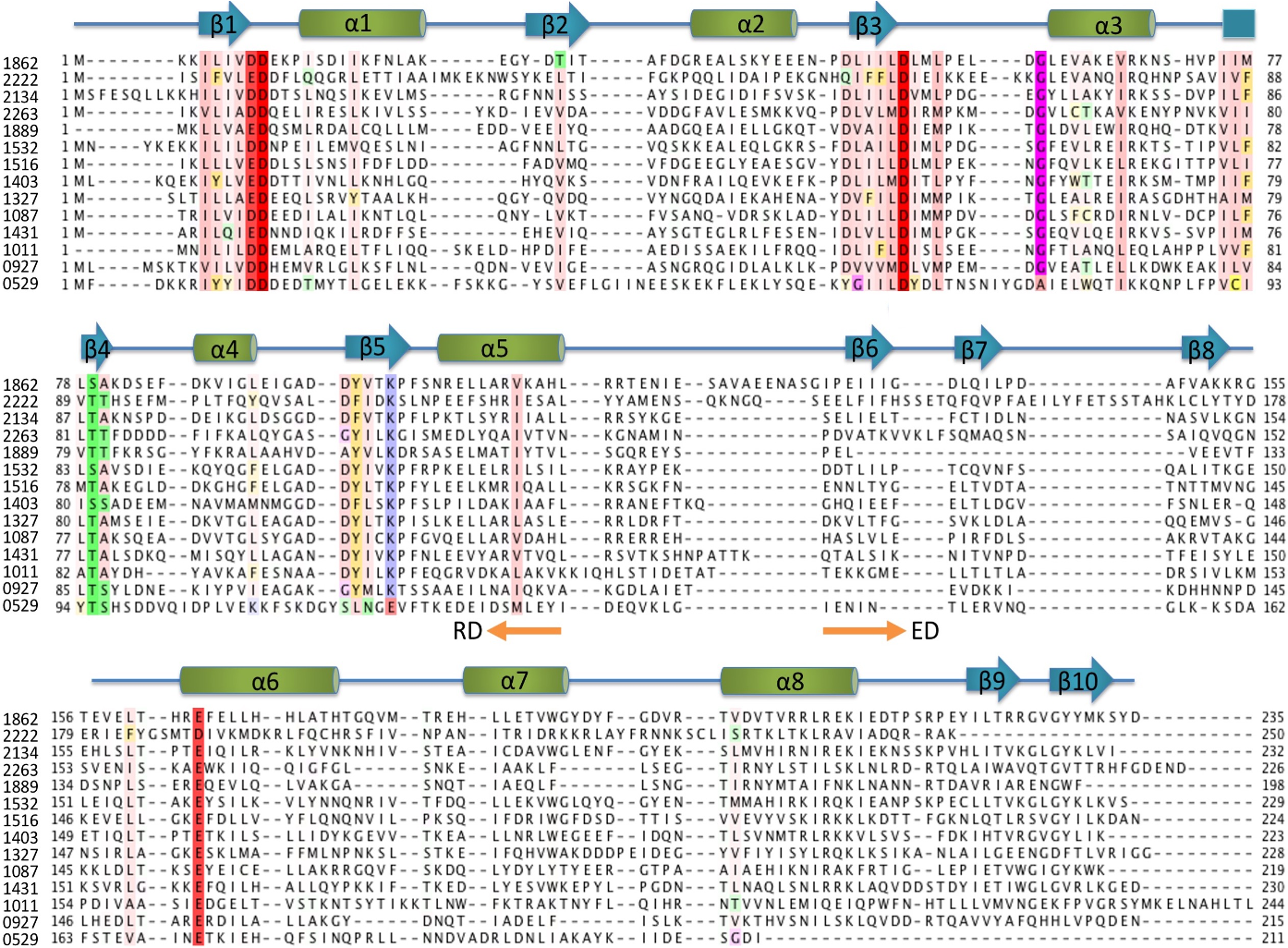


B


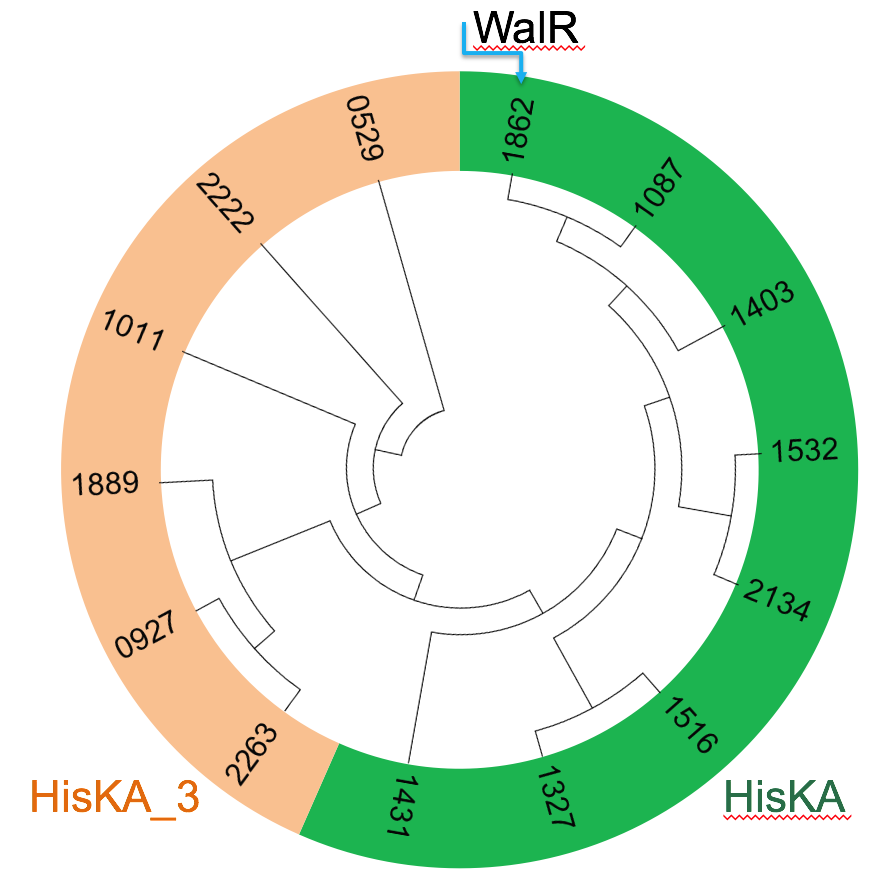


Figure S2. Sequence alignment of 14 RRs in *S. mutans*.

(A) Full-length RR sequences were aligned in PROMALS3D (Pei et al., 2008). Residues are colored progressively by their conservation in Jalview (Waterhouse et al., 2009). (B) Phylogenetic analysis of *S. mutans* RRs. Evolutionary relationship of RRs is shown in a circular tree, which are grouped based on the HisKA conservation of their cognate HKs colored in green and orange. RRs are named with four digits in their protein ID: NP_72****.1.

Supplementary figure 3


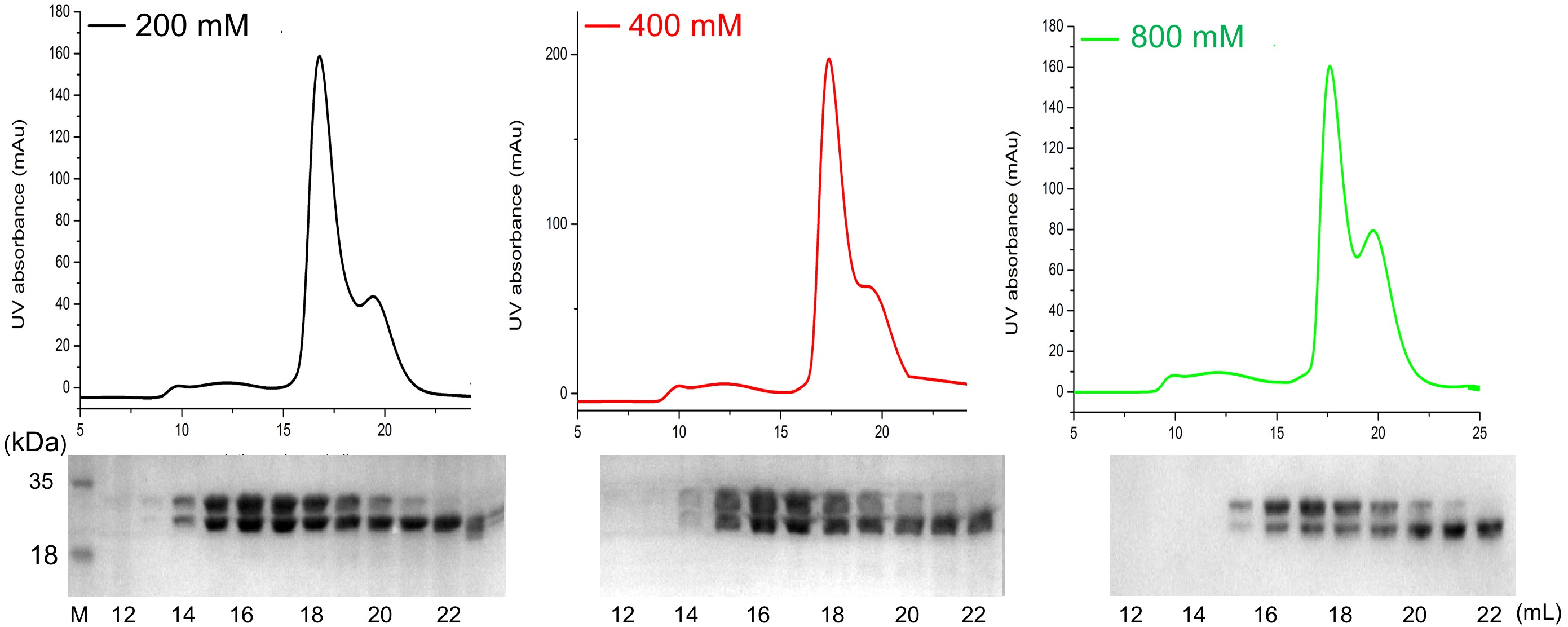


Figure S3. Stability analysis for the WalK (196-450) complexes with full-length WalR. The stability was estimated by gel filtration on Superdex S200 with increased NaCl concentrations in a basic buffer of 20 mM Tris-HCl, pH 8.0, 1 mM EDTA and 1 mM DTT. Three aliquots of the complexes were mixed with 1:1.3 WalK:WalR. The fractions were analyzed in 15% w/v SDS-PAGE, and stained by CBB as shown in the analysis below each chromatogram. Elution volumes were shown below each panel.

Supplementary figure 4


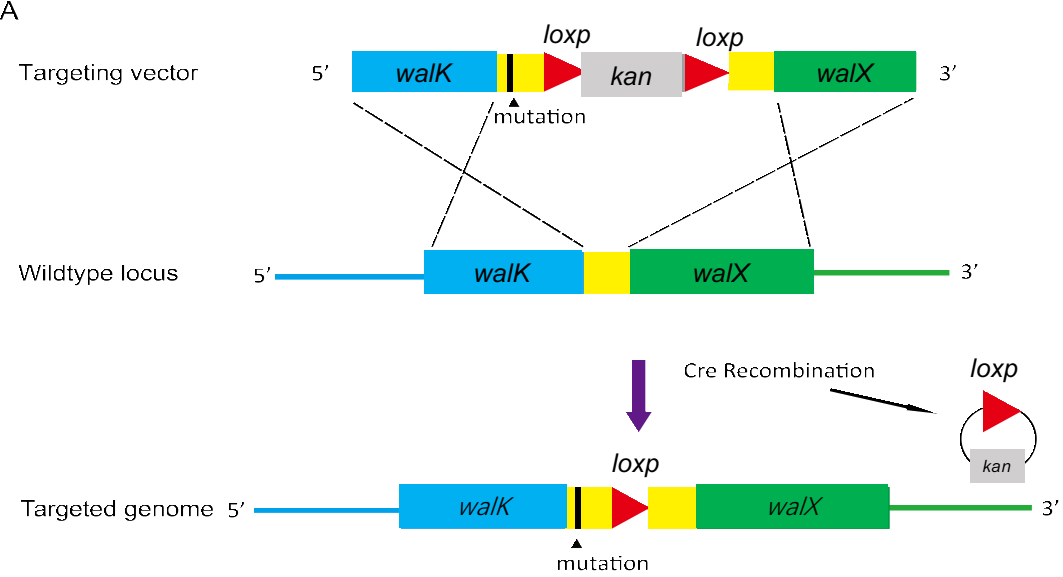


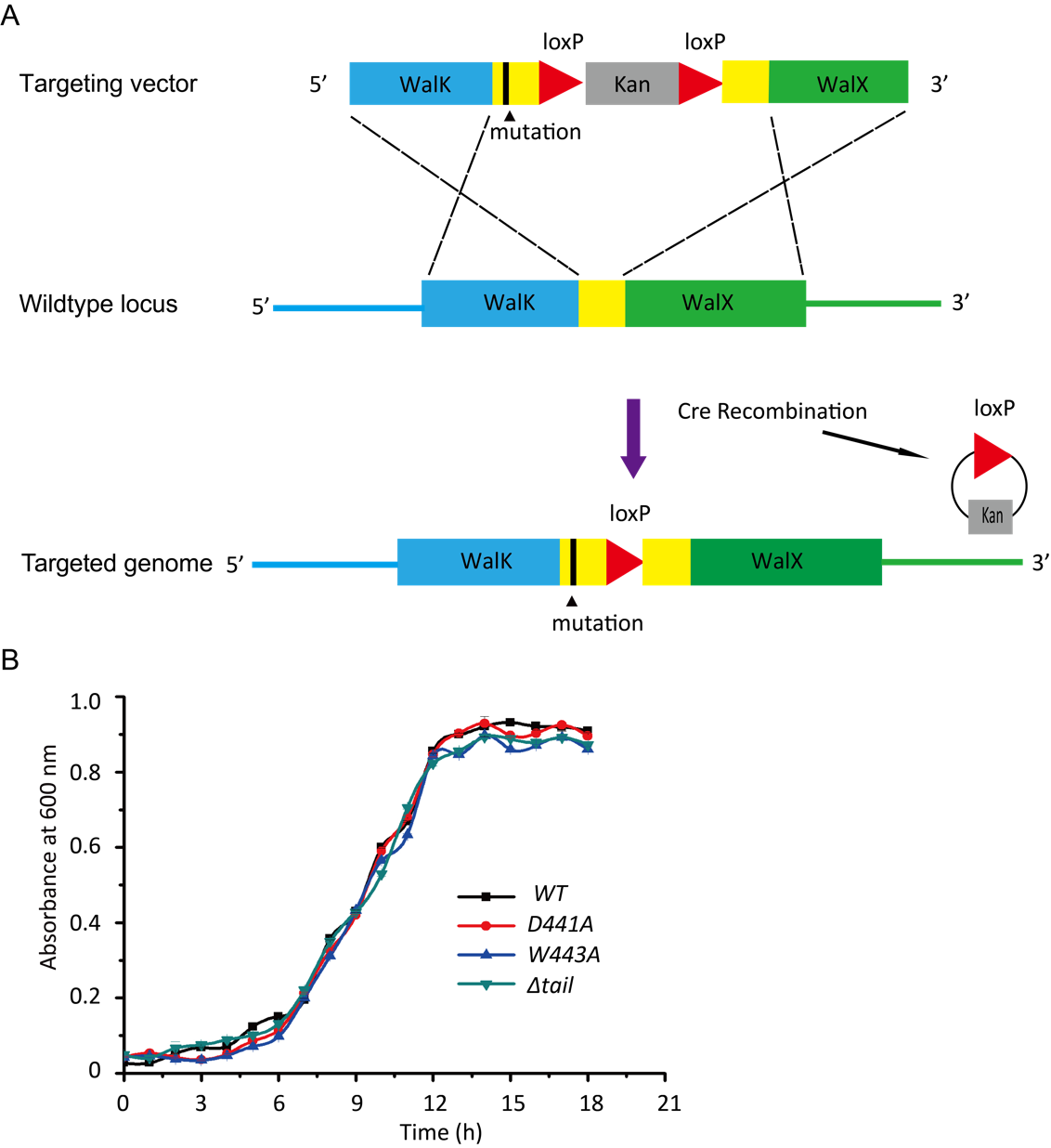


Figure S4. Construction of *S. mutans* mutants.

(A) Targeted mutagenesis in the *S. mutans* genome using Cre-loxP as described in methods. (B) Growth curves of *S. mutans* strains. The strains were recovered overnight and inoculated in fresh media under anaerobic conditions. The OD_600_ was measured until the bacteria grew to stationary phase. All data points were averaged from three independent experiments.

Supplementary figure 5


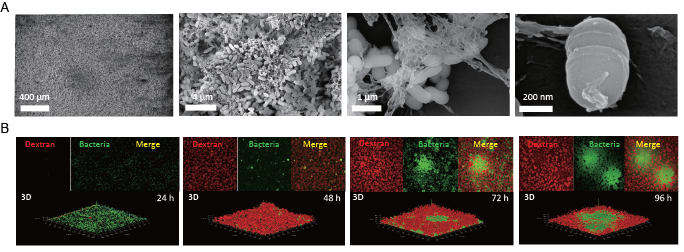


Figure S5. Biofilm analysis of *S. mutans* D441A strain.

(A) SEM analysis of mature biofilms grown for 72 h. (B) Biofilm development. The biofilms were labeled with red fluorescent dextran for EPS and stained with green fluorescent CYTO 9 for total bacteria. The 3D images were constructed using the ZEN software package.

Supplementary figure 6


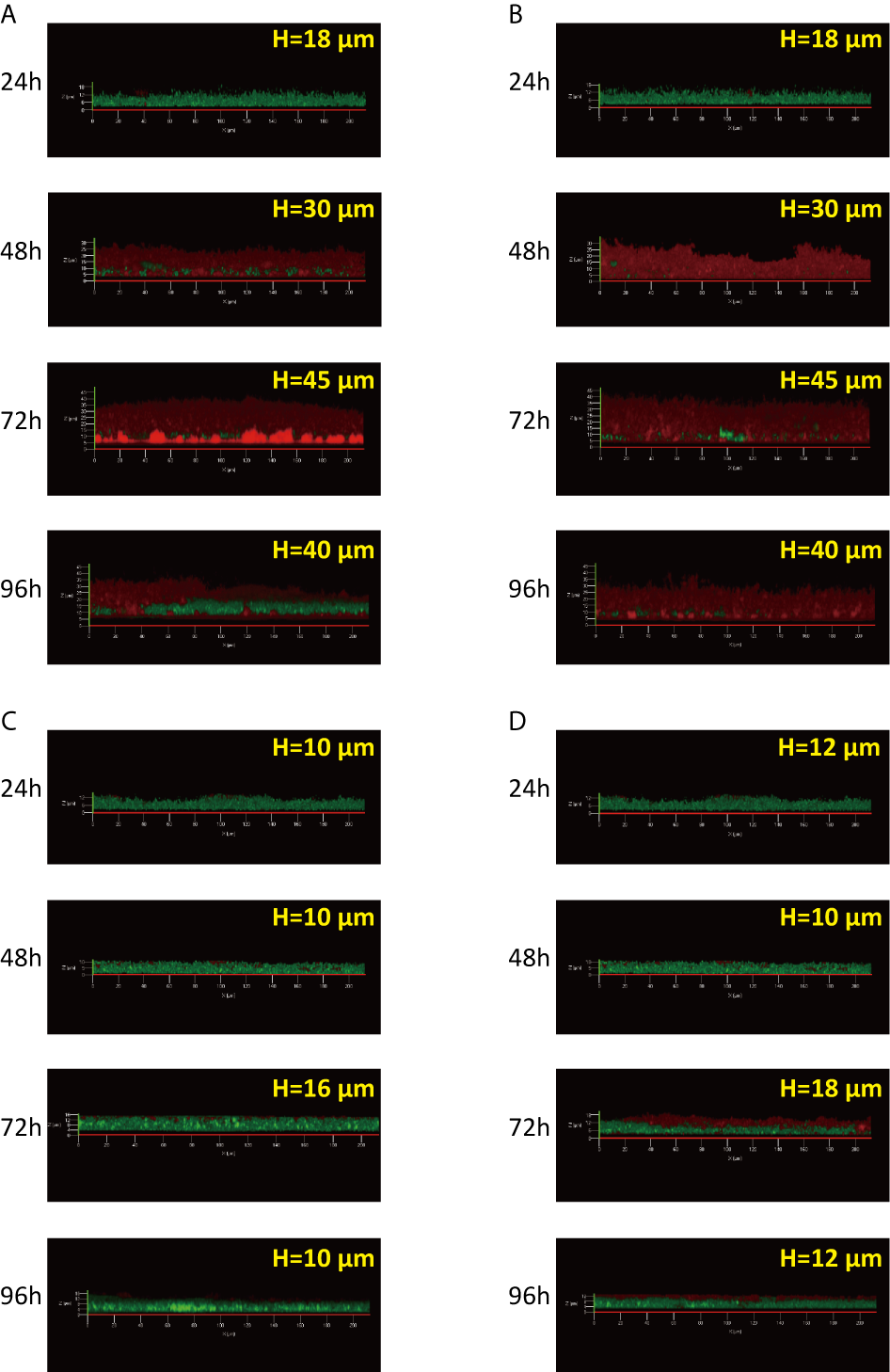


Figure S6. The averaged biofilm thickness of *S. mutans* strains *WT* (A), D441A (B), W443A (C) and Δtail deletion (D). The biofilms were labeled with red fluorescent dextran for EPS and stained with green fluorescent CYTO 9 for total bacteria. All images were constructed using the ZEN software package.

Supplementary figure 7


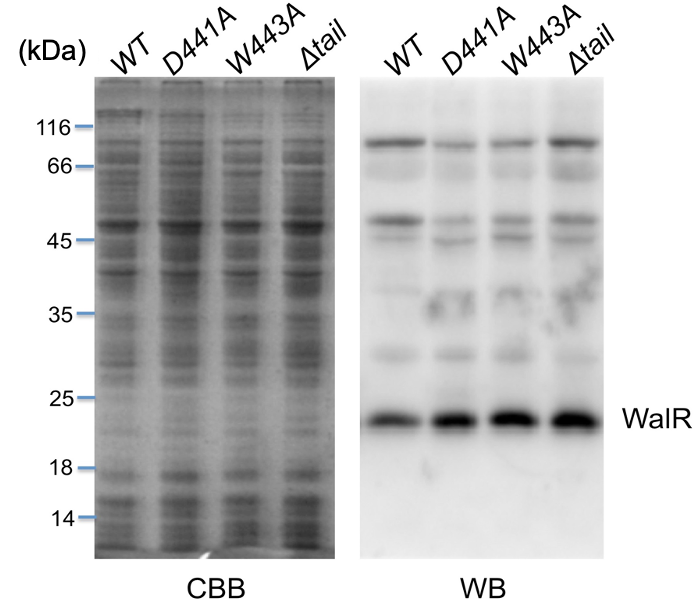


Figure S7. Efficiency of anti-WalR antibody developed in rabbits. The *S. mutans* biofilms were collected at 48 h and the total proteins were prepared as described in the mass spectroscopy section. The left panel shows proteins separated by 15% w/v SDS-PAGE and stained by CBB. The right panel shows a western blot using the WalR antibody diluted to 1:1000.

Supplementary Table 1

Table S1. Top proteins that were significantly altered in the *S. mutans* *Δtail* deletion strain.

| Δtail/WT | **Code** | **Name** | **Function** |
| --- | --- | --- | --- |
| -3.9 | Q8CVC7 | Gps40 | Putative 40K cell wall protein precursor, may regulate cell morphology |
| -3.2 | Q8DW79 | unknown | 86 amino acid |
| -3 | Q54443 | DexA | Dextranase, alpha-1,6-glucan-6-glucanohydrolase, sucrose-independent adherence to surface |
| -2.8 | Q8DW70 | unknown | 90 amino acids |
| -2.8 | P23504 | SpaP | Cell surface antigen I/II, localized on cell wall, important role in initial attachment to tooth surfaces |
| -2.7 | P13470 | GtfC | Glucosyltransferase-SI for water-insoluble glucan synthesis, key in dental plague development |
| -2.4 | Q8DS34 | IF-1 | Essential components for the initiation of protein synthesis. |
| -2.4 | Q8DTE3 | Cas9 | CRISPR-associated endonuclease Cas9 |
| -2.2 | Q8DVC8 | unknown | 76 amino acids |
| -2.1 | Q8DV79 | ComEC | Putative metal-dependent hydrolase, beta-lactamase superfamily II for competence |
| -1.9 | P08987 | GtfB | Glucosyltransferase-I for water-insoluble glucans synthesis |
| -1.7 | Q8DUX2 | unknown | 70 amino acids |
| -1.7 | Q8DUC8 | GntR | GntR-like transcriptional regulator with a H-T-H DNA binding domain |
| -1.6 | Q8DTF1 | GbpC | Glucan-binding protein C |
| -1.4 | Q8DUE5 | DapA | 4-hydroxy-tetrahydrodipicolinate synthase |
| -1.3 | P10539 | DhaS | Aspartate-semialdehyde dehydrogenase |
| -1.3 | Q8DSJ6 | YajC | Preprotein translocase subunit |
| -1.2 | Q8DW95 | PemK | Putative ppGpp-regulated growth inhibitor, PemK-like, MazF-like toxin of type II toxin-antitoxin system |
| -1.2 | I6L910 | PtxB | Putative phosphoenolpyruvate/sugar phosphotransferase (PTS) system, enzyme IIB subunit |
| -1 | Q8DUB9 | PycB | Putative pyruvate carboxylase/oxaloacetate decarboxylase, alpha subunit |
| -1 | Q8DU84 | ProV | ABC-type proline/glycine betaine transport system, ATP-binding protein |
| -1 | Q8DW65 | unknown | 103 amino acids |
| -1 | Q8DUC2 | CitE | Citrate lyase, beta subunit |
| 1 | Q8DSC2 | PtnD | Putative PTS system, mannose-specific component IID |
| 1 | Q8DV56 | TauB | ABC-type nitrate/sulfonate/bicaronate transporter system, ATPase subunit |
| 1 | Q8DUA2 | CcmA | ABC-type multidrug transporter system, ATPase subunit |
| 1 | Q8DSL7 | Cas5I | Endonuclease, maintenance of CRISPR repeat elements |
| 1.2 | P49331 | GtfD | Glucosyltransferase-S, synthesizes water-soluble glucans |
| 1.2 | Q8DTJ0 | FtsXL | FtsX-like permease |
| 1.3 | Q8DVM0 | HisM | Putative amino acid ABC transporter permease |
| 1.3 | Q8DUG7 | CaiA | Acyl-CoA dehydrogenase, flavin adenine dinucleotide binding |
| 1.3 | Q8DTC0 | CesA | Putative glycosyltransferase, catalyzing the elongation of polysaccharide chains. |
| 1.4 | Q8DU79 | unknown | Putative ABC transporter, ATP-binding protein |
| 1.4 | Q8DV57 | TauA | Putative ABC-type nitrate/sulfonate/bicaronate transporter system, substrate-binding protein |
| 1.5 | Q8DSC3 | PtnC | Putative D-glucosamine PTS permease, mannose-specific component IIC |
| 1.6 | Q59939 | CitZ | Citrate synthase in tricarboxylic acid cycle |
| 1.7 | Q8DSN3 | Acp | 74 amino acid, acyl carrier protein of the growing fatty acid chain in fatty acid biosynthesis |
| 1.8 | Q8DUE3 | Smp35 | Integral component of membrane, 321 amino acids, funcationally unknown |
| 2.4 | Q8DVS0 | CppA | Putative C3-glycoprotein degrading proteinase, involved in pathogenicity |

Note: The difference between Δtail/*WT* was determined by a formula $Log$_2_$[LFQ\left( \frac{\Delta tail}{WT} \right)]$.

Supplementary Table 2

Table S2. Primers used in the experiments

| **Name** | **Sequence** |
| --- | --- |
| *S. mutans walK* 31-450 Forward | AGA **GGATCC** AATTACAGAGAGTATAAAAATAATAATCAAGTTAAAC |
| *S. mutans walK* 31-450 Reverse | AGA **AAGCTT** TCATGATTCGTCTTCATCTTCTTCC |
| *S. mutans walK* 196-450 Forward | AGA **AAGCTT** TTACATGATGCGACCGAACAGG |
| *S. mutans walK* 196-450 H217A1 | GTCAGTGCGGAACTGCGGACACCATTAAC |
| *S. mutans walK* 196-450 H217A2 | TTCCGCCTTTGTTTCTAATGTCAGTCAT |
| *S. mutans walK* 196-450 D441A1 | GCAATTGCGGAATGGGAAGAAGATG |
| *S. mutans walK* 196-450 D441A2 | TTCCGCAATTGCATCATTATCGTTTTC |
| *S. mutans walK* 196-450 W443A1 | GATGAAGCGGAAGAAGATGAAGACG |
| *S. mutans walK* 196-450 W443A2 | TTCCGCTTCATCAATTGCATCATTATCG |
| *S. mutans* w*alK* 196-450 ∆tail Reverse | AGA **AAGCTT** ATCGTTTTCATAAGGTAAAACGATG |
| *S. mutans walR* FL Forward | AGA **GGATCC** ATGAAGAAAATTCTAATCGTTGACG |
| *S. mutans walR* FL Reverse | AGA **GCTAGC** TTAGTCATATGATTTCATGTAATAACCAAC |
| *S. mutans* *walR* RD Reverse | AGA **GCTAGC** TTCAGGTATACCTGAAGCATTTTC |
| *S. mutans* *walR* DBD Forward | AGA **GGATCC** TTCAGGTATACCTGAAGCATTTTC |
| *S. aureus walK* 364-608 Forward | AGA **GGATCC** CATGACGTAACTGAACAACAAC |
| *S. aureus walK* 364-608 Reverse | AGA **GCTAGC** TTA TTC ATC CCA ATC ACC GTC |
| *S. aureus walK* 364-608 D605A Reverse | AGA **GCTAGC**TTATTCATCCCACGCACCGTCTTCAATGACTTCAC |
| *S. aureus walK* 364-608 D607A Reverse | AGA **GCTAGC** TTATTCATCCGCATCACCGTCTTCAATGACTT |
| *S. aureus* *walK* 364-608 D607A Reverse | AGA **GCTAGC** TTATTCCGCCCAATCACCGTCTTCAATGA |
| *S. aureus* *walK* 364-608 ∆tail Reverse | AGA **GCTAGC** TTACAATGACTTCACATGGAAGTG |
| *S. aureus* *walR* FL Forward | GAC **GGATCC** ATGGCTAGAAAAGTTGTTGTA |
| *S. aureus* *walR* FL Reverse | AGA **GCTAGC** TTACTACTCATGTTGTTGGAGG |
| RT-PCR |  |
| *gbpA Forward* | GATGGATCCCATTGAAAAAGATAATGGCTTC |
| *gbpA Reverse* | GATCTCGAGTTTACCTCCTTAAAAAATAGTAATC |
| *gbpB Forward* | *GATGGATCCATTGACAGCTTATCCTTTAAATG* |
| *gbpB Reverse* | GATCTCGAGTACAAATATAACTCCTTTTTCGAT |
| *gbpC Forward* | GATGGATCCCTATGATATCAGTTTAGACTC |
| *gbpC Reverse* | GATCTCGAGAAAAACCATCCTTTATATTATTAATT |
| *gtfB Forward* | GATGGATCCCGACAGCAATTAGACTGTTG |
| *gtfB Reverse* | GATCTCGAGTAGGAACCTCCAAATTTTAAAC |
| *gtfC Forward* | GATGGATCCGGAGAACGAGTTCGGATTAAC |
| *gtfC Reverse* | GATCTCGAGATTTCCTCCAAAAATAGTTAGAG |
| *gtfD Forward* | GATGGATCCGTGTTAAAAGATGTAATTTATAGC |
| *gtfD Reverse* | GATCTCGAGAAAATATCCTCCTTTATCAGTTC |
| *16S rRNA* *Forward* | ACCAGAAAGGGACGGCTAAC |
| *16S rRNA Reverse* | TAGCCTTTTACTCCAGACTTTCCTG |

Note: Enzyme restriction sites are in bold letters.
